# Enhanced bioproduction and processing of mandelic acid enantiomers: towards a sustainable platform for high-value pharmaceutical and polymer applications

**DOI:** 10.1186/s13068-025-02727-1

**Published:** 2025-12-17

**Authors:** Hanan Latif Messiha, Alec Banner, Mohamed Amer, Christopher James Robinson, Aula Alwattar, Viranga Tilakaratna, Rosalind Le Feuvre, Nigel Shaun Scrutton

**Affiliations:** 1 Future Biomanufacturing Research Hub (Future BRH), Manchester Institute of Biotechnology (MIB), 131 Princess Street, Manchester, M1 7DN UK; 2 Department of Chemistry, School of Natural Sciences, Faculty of Science and Engineering, University of Manchester, Oxford Road, Manchester, M1 9PL UK

**Keywords:** Mandelic acid (MA), (*R*)-Mandelic acid, (*S*)-Mandelic acid, Metabolic engineering, Microbial fermentation, Sustainable biomanufacturing, Biotechnology platform, Sustainable biopolymers, Polymandelide (PM), Mandelic acid condensation polymer (SAMMA, PPCM)

## Abstract

**Background:**

Mandelic acid (MA) is a high-value chiral platform molecule with broad applications in pharmaceutical synthesis, cosmetic formulations, and polymer production. Conventional chemical synthesis is limited by harsh reaction conditions, poor enantioselectivity, and environmental concerns. Microbial biosynthesis offers a sustainable and stereoselective alternative; however, its industrial application is constrained by low titres, suboptimal productivity, and inefficient downstream recovery. This study reports an engineered microbial chassis that enables enhanced biosynthesis of MA enantiomers with integrated downstream compatibility.

**Results:**

The biosynthetic potential of *Escherichia coli* was harnessed through targeted metabolic engineering and pathway optimisation for the biosynthesis of (*R*)- and (*S*)-MA. Batch fermentations in rich medium produced 1.6 g/L (*R*)-MA and 1.8 g/L (*S*)-MA. Transitioning to fed-batch cultivation in defined minimal medium, under non-optimised conditions, increased titres to 2.9 g/L; *ee* = 99% for (*R*)-MA and 5.7 g/L; *ee* = 93% for (*S*)-MA, representing the highest reported in vivo titres of MA enantiomers achieved in *E. coli* to date. A two-step downstream process comprising solvent extraction and crystallisation enabled the recovery of MA at high purity (> 99.0%), with recovery efficiencies of 84% for (*S*)-MA and 77% for (*R*)-MA. To validate the functional utility of bio-based MA, SAMMA, a sulfuric acid condensation polymer with documented antiviral and contraceptive properties, was synthesised from both bio-based and commercial MA. Additionally, mandelide, a monomer precursor for the biodegradable polystyrene analogue polymandelide (PM), was synthesised to illustrate the platform’s relevance to sustainable polymer applications.

**Conclusions:**

This study establishes a robust proof of concept for a microbial platform enabling enantioselective MA biosynthesis from renewable carbon sources. Through the integration of metabolic engineering, downstream process development and application-driven validation, this platform lays the foundation for a scalable and industrially relevant bioproduction strategy. Aligned with the principles of green chemistry and the circular bioeconomy, this approach offers a sustainable and environmentally responsible route to high-value chiral chemicals.

**Supplementary Information:**

The online version contains supplementary material available at 10.1186/s13068-025-02727-1.

## Background

The global transition towards sustainable and eco-friendly manufacturing has accelerated the exploration of microbial bioeconomy to replace fossil fuel-derived products with renewable, bio-based alternatives [1, 2]. Advances in metabolic engineering and synthetic biology have significantly expanded the capabilities of microbial platforms to convert low-cost, renewable feedstocks into a diverse range of high-value bioproducts. However, challenges such as limited strain performance, process scalability, and downstream recovery issues continue to impede industrial implementation [3]. Addressing these limitations is critical to unlocking the full potential of bio-based production systems. In this context, the diversification of microbial biosynthesis to expand the portfolios of bio-based chemicals has emerged as a strategic priority in industrial biotechnology [4].

**Fig. 1:**
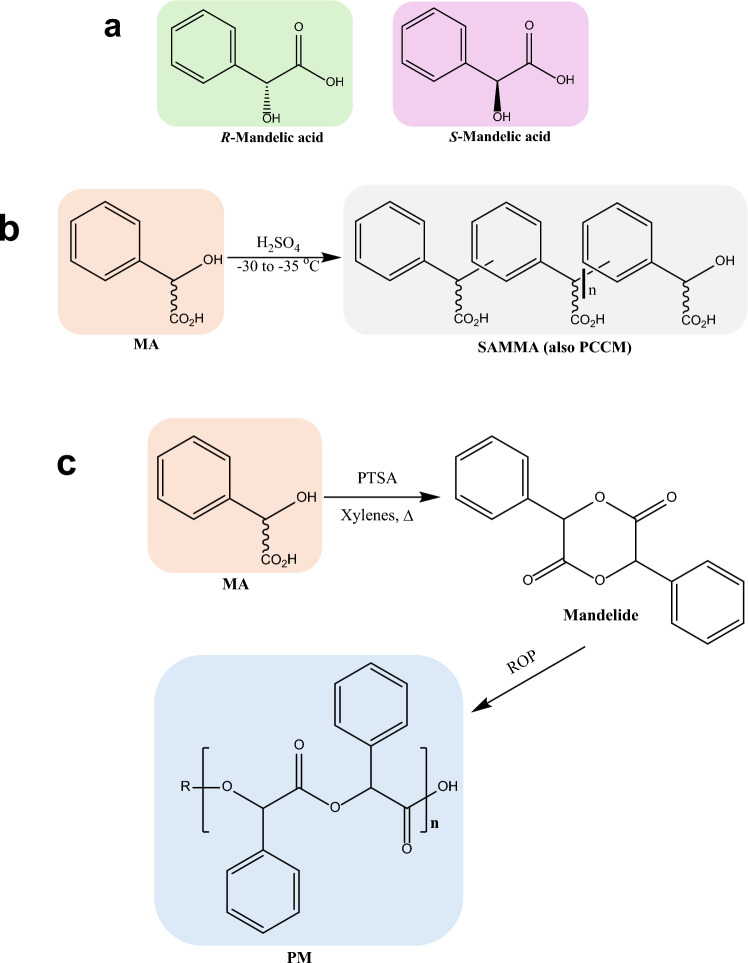
Mandelic acid (MA) enantiomers and examples of MA-derived polymers. **a** Enantiomers of MA. **b** Sulfuric acid-modified mandelic acid (SAMMA) polymer; or polyphenylene carboxymethylene (PPCM). **c** Polymandelide (PM) synthesised by ring-opening polymerisation (ROP) of mandelide. (PTSA, *p*-toluenesulfonic acid)

Among high-value chemicals, mandelic acid (MA), a chiral α-hydroxy acid, stands out as one such compound with applications spanning the cosmetics, pharmaceuticals, and polymer sectors. MA is extensively incorporated into dermatological and cosmetic formulations due to its clinically validated exfoliative, antimicrobial and antioxidant properties. Beyond its established uses in dermatology, MA serves as a key intermediate in the synthesis of active pharmaceutical ingredients [5, 6] and its enantiomers (Fig. 1) are widely utilised as chiral auxiliaries in stereoselective syntheses [7–9]. MA is also a versatile building block in the synthesis of various polymers and copolymers, serving as a key monomer for next-generation biodegradable polymers. One example is polymandelide (PM), a biodegradable polyester derived via ring-opening polymerisation of mandelide (Fig. 1), with thermal and mechanical properties comparable to polystyrene, making it suitable for packaging and biomedical applications [10–13]. Another MA-derived polymer, polyphenylene carboxymethylene (PPCM, commonly known as sulfuric-acid-modified mandelic acid, SAMMA; Fig. 1), possesses potent antiviral and contraceptive properties [14–18]. Additionally, MA-derived copolymers have been investigated for various biomedical applications including drug delivery [19, 20], tissue engineering and implantable biomaterials [21], underscoring MA’s industrial significance and its potential for sustainable material innovation.

The global MA market was valued at approximately USD 291.3 million in 2024 and is projected to reach USD 782.6 million by 2033, reflecting its growing commercial importance [22].

Traditionally, MA is obtained either through the hydrolysis of amygdalin, a cyanogenic glycoside present in bitter almonds or via chemical synthesis from petroleum-derived intermediates. Industrial synthesis predominantly involves the hydrolysis of mandelonitrile [23], a process that raises significant environmental and safety concerns, uses toxic reagents, and requires an extensive, costly purification process to isolate the enantiomers in pure forms. Given the distinct pharmacological properties and target-specific applications of each MA enantiomer, there is a growing demand for enantiomerically pure products. As a result, chemoenzymatic strategies have been developed to achieve enantioselective synthesis. However, these approaches are often constrained by complex multi-step reaction sequences, substrate and intermediate toxicity, limited scalability, and suboptimal overall efficiency [24–26].

Over the past two decades, biotechnological innovations, particularly microbial biotransformation and enzymatic catalysis employing native or engineered microbial systems, have emerged as promising alternatives for the sustainable production of MA. For example, (*R*)-MA has been produced via biotransformation of mandelonitrile and mandelamide [27–29], and both (*R*)- and (*S*)-MA have been synthesised through whole-cell cascade transformations from substrates such as *L*-phenylalanine, glucose, glycerol and styrene [30, 31]. Despite these advances, persistent challenges, including low product titres, precursor toxicity, and the accumulation of undesirable byproducts, continue to limit industrial feasibility.

Recent progress in both chemoenzymatic and microbial bioconversion strategies has been notable [32]; however, the de novo microbial biosynthesis of MA enantiomers directly from renewable feedstocks remains relatively underexplored, with reported titres generally falling below 1 g/L [33–36]. In contrast, significant progress has been made in the microbial production of racemic MA, with a recent study reporting a titre of 9 g/L in a 5-L fed-batch bioreactor in rich medium [37]. This highlights the need for more efficient microbial platforms capable of enantioselective MA production under conditions relevant to industrial applications.

In this study, we present an integrated biomanufacturing platform designed to enhance the sustainable microbial production of MA enantiomers. The primary goal is to develop an improved microbial platform for the enantioselective biosynthesis and recovery of MA, a versatile precursor in fine chemicals, polymers, and pharmaceuticals. This platform builds on *E. coli* chassis strains previously constructed in our laboratory [35], incorporating systematic redesigns implemented to enable enhanced flux towards MA biosynthesis. Beyond strain engineering, this platform also integrates a streamlined downstream processing to enable efficient product recovery. While the central focus of this work is biosynthesis and product recovery, limited downstream application studies were incorporated in this study to demonstrate that the bio-produced enantiomers are compatible with established high-value applications, highlighting the platform’s potential for industrial biotechnology.

## Methods

All chemicals, reagents, and solvents used in this study were obtained from commercial laboratory suppliers and were of high analytical grade.

### Plasmids, primers, bacterial strains, and growth conditions

A comprehensive list of all plasmids, primers, and bacterial strains used or generated in this study can be found in Table S1–S3. Genes were synthesised using either the GeneArt gene synthesis service (Thermo Fisher Scientific) or Twist Bioscience. DNA primers were purchased from Integrated DNA Technologies. All plasmids and bacterial strains constructed for this study were sequenced by Plasmidsaurus. Detailed procedures for the construction of gene expression plasmids, gene knockout plasmids, engineered *E. coli* strains, competent cells preparation, and transformation procedures are provided and described in the Supplementary Information.

### Construction of engineered* E. coli* strains

The engineered strains used in this study are derivatives of *E. coli* DH5α: SBC010793, carrying deletions in *tyrR*, *tyrA*, and *lacZ*::8376 loci [35], and SBC015953, further engineered to include a *tyrB* deletion (this study). Further details are described and can be found in the Supplementary Information. Expression plasmids encoding 4-hydroxymandelate synthase (HMAS) variants from *Streptomyces yokosukanensis—Sy*HMAS(S204V) for (*R*)-MA production and *Sy*HMAS (I219V) for (*S*)-MA—were introduced into these strains by transformation (as described in the Supplementary Information), either individually or in combination with the *L*-amino acid deaminase (*Pm*LAAD), to construct six recombinant strains (Table 1). *Pm*LAAD is an engineered *L*-amino acid deaminase from *Proteus mirabilis* [41] employed in this study for the purpose of improving the flux towards phenylpyruvate, the precursor for MA biosynthesis. These six recombinant strains were designated as MA-1 through MA-6 (Table 1) for simplicity thereafter. Strains were preserved as glycerol stocks and stored at − 70 °C.

**Table 1:** Recombinant *E. coli* strains engineered for the production of MA enantiomers

Recombinant strain	Genotype	Plasmid
MA-1	DH5α *ΔtyrR ΔtyrA lacZ*::8376 SBC010793	*Sy*HMAS(S204V) SBC010825
MA-2	DH5α *ΔtyrR ΔtyrA lacZ*::8376 SBC010793	*Sy*HMAS(I219V) SBC010826
MA-3	DH5α *ΔtyrR ΔtyrA ΔtyrB lacZ*::8376 SBC015953	*Sy*HMAS(S204V) SBC010825
MA-4	DH5α *ΔtyrR ΔtyrA ΔtyrB lacZ*::8376 SBC015953	*Sy*HMAS(I219V) SBC010826
MA-5	DH5α *ΔtyrR ΔtyrA ΔtyrB lacZ*::8376 SBC015953	*Sy*HMAS(S204V) + *Pm*LAAD SBC015937
MA-6	DH5α *ΔtyrR ΔtyrA ΔtyrB lacZ*::8376 SBC015953	*Sy*HMAS(I219V) + *Pm*LAAD SBC015938

Six recombinant strains (MA-1 to MA-6) were generated by transforming engineered *E. coli* strains SBC010793 and SBC015953 with plasmids harbouring either *Sy*HMAS variants alone (strains MA-1 – MA-4) or *Sy*HMAS variants with *Pm*LAAD (strains MA-5 and MA-6). The SBC numbers displayed are catalogue numbers from our inventory system for strains and plasmids (see Tables S1 and S3). The 8376-construct inserted at the *lacZ* locus is an 8.7 kb sequence carrying the *pheA*(G309C), *ppsA*, *aroF*(P148L) and *tktA, ppsA, aroF(P148L) and tktA* genes

### Screening of recombinant strains for in vivo MA production

Pre-cultures of MA-1 through MA-6 were initiated by inoculating each strain into 5 mL of buffered Terrific Broth (TB) medium (pH 7.0) supplemented with 0.8% (w/v) glycerol and 100 µg/mL carbenicillin. Cultures were incubated overnight at 30 °C with agitation (180 rpm). The overnight cultures were diluted 1:100 into 50 mL of fresh TB medium (supplemented as above) in 250-mL shaking flasks and incubated at 30 °C with shaking (180 rpm) until the cultures reached mid-exponential phase (optical density, OD_600_ ≈ 1.0–1.5). Gene expression was then induced by adding 0.1 mM isopropyl *β*-D-1-thiogalactopyranoside (IPTG). Cultures were subsequently incubated at 30 °C under the same conditions for 72 h. The supernatants were harvested by centrifugation, filtered and analysed for MA content by high-performance liquid chromatography (HPLC) as described in the analytical methods section and Supplementary Information.

### Fermentation

Batch fermentations were conducted in 200 mL volumes of TB medium supplemented with 0.8% (w/v) glycerol and 100 µg/mL carbenicillin in shaking flasks at 30 °C. Gene expression was induced at OD_600_ ≈ 1.0–1.5 by the addition of 0.1 mM IPTG. Fed-batch fermentations were performed in 150 mL volumes of defined minimal medium, supplemented with carbenicillin per bioreactor vessel in a modular Sartorius AMBR250 bioreactor system (Ambr® 250 modular) with a total working volume of 200 mL per vessel (including 50 mL feed). The fed-batch fermentations were conducted at 30 °C throughout the entire fermentation run and maintained at 30% dissolved oxygen (DO) via an automated cascade control system, initially adjusting agitation speed (300 – 2500 rpm), followed by aeration rate (1 -2.5 vvm), in response to DO fluctuations. pH was maintained at 7.0 by the automatic addition of 15% (v/v) NH_4_OH or 2 M HCl, as required. Prior to each fermentation run, DO and pH probes were calibrated according to the manufacturer’s guidelines. Polypropylene glycol was added as an antifoam agent at a 1 in 10,000 dilution, with additional 10 µL doses administered every 12 h. Carbenicillin was supplemented at 72 h and 144 h with top-up doses of 50 µg/mL. The composition of the defined minimal medium per L was as follows: 0.015 g calcium chloride dihydrate (CaCl_2_.2H_2_O), 3 g potassium dihydrogen phosphate (KH_2_PO_4_), 12 g dipotassium hydrogen phosphate (K_2_HPO_4_), 5 g ammonium sulfate ((NH_4_)_2_SO_4_), 0.075 g ferrous sulfate heptahydrate (FeSO_4_.7H_2_O), 1 g trisodium citrate dihydrate (C_6_H_5_Na_3_O_7_.2H_2_O), 4 g yeast extract, 3 mL of 100 g/L magnesium sulfate heptahydrate (MgSO_4_.7H_2_O) solution, 1 mL of trace element solution, 1 mL of 5 g/L thiamine–HCl solution, 8 g glycerol and 0.3 g tyrosine. The trace element stock solution was prepared with the following composition per L: 2 g aluminium sulfate octadecahydrate (Al_2_(SO_4_)_3_∙18 H_2_O), 0.8 g cobalt sulfate heptahydrate (CoSO_4_∙7H_2_O), 2.5 g copper(II) sulfate pentahydrate (CuSO_4_∙5 H_2_O), 0.5 g boric acid (H_3_BO_4_), 24 g manganese(II) sulfate monohydrate (MnSO_4_∙H_2_O), 3 g sodium molybdate dihydrate (Na_2_MoO_4_∙2H_2_O), 31.5 g nickel(II) sulfate hexahydrate (NiSO_4_∙6H_2_O), 15 g zinc sulfate heptahydrate (ZnSO_4_∙7H_2_O) and 2.4 mL of 25% sulfuric acid (H_2_SO_4_). Feeding was commenced upon the depletion of the initial glycerol, as indicated by a ‘spike/sharp increase’ in DO (> 40%). The feed solution consists of 300 g glycerol, 20 g yeast extract, 0.1 mM IPTG and 100 µg/mL carbenicillin per L. Feed rate was initially set at 0.15 mL/h and increased exponentially at a rate of 0.15 h^−1^. Once the feed rate reached 5 mL/h, it was maintained at this rate until a total of 50 mL of feed was administered. Samples (2 mL) were aseptically collected at various time points for monitoring growth and product formation. Cell pellets were harvested by centrifugation at 14,400 × rpm for 5 min. The resulting supernatants were filtered through 0.22 µm membranes and analysed to monitor MA production and glycerol consumption by HPLC, as described in the analytical methods section of the Supplementary Information. Data are presented as mean ± standard deviation from replicate experiments.

### Downstream processing for the isolation of MA enantiomers

Recovery and purification of MA enantiomers from the fed-batch fermentation broth were performed with modifications to the protocol described by Zang et al. [38, 39]. Upon completion of fermentation, the broth was subjected to centrifugation at 6,000 rpm for 15 min at 4 °C, with the process repeated twice to ensure efficient removal of biomass. The resulting supernatant was treated with 1% (w/v) Celite^®^ 454 and stirred at room temperature for 5 min to facilitate the removal of fine particulates, followed by vacuum. The pH of the clarified filtrate was adjusted to 10.0 by the gradual addition of 10 M sodium hydroxide (NaOH) under continuous stirring. The alkaline solution was then subjected to liquid–liquid extraction with butyl acetate (aqueous:organic, 2:1 v/v) for 15 min at room temperature. The aqueous phase, containing the sodium salt of MA, was retained and treated with 1% (w/v) activated carbon (NORIT® A SUPRA) at 40 °C with agitation (150 rpm) for 30 min. Carbon particulates were then removed by vacuum filtration. Following decolourisation and clarification, the pH was adjusted to pH 2.5 by the controlled addition of concentrated H_2_SO_4_ (95%) under stirring at room temperature. The acidified solution was extracted twice with butyl acetate (aqueous:organic, 1:2 v/v), each extraction lasting 30 min at room temperature. The combined organic phases were concentrated to dryness under reduced pressure using a rotary evaporator (Rotavaor^®^ R-300, BUCHI, Switzerland) at 45 °C, 30 mbar and 180 rpm, yielding a crude MA residue. The butyl acetate was recovered for potential reuse. Initial characterisation of the crude product was performed by nuclear magnetic resonance (NMR) spectroscopy, HPLC and infrared (IR) spectroscopy as described in the Supplementary Information. To enhance higher purity, the crude MA was dissolved in 20 mL deionised water, acidified to pH 2.5 as previously described, and re-extracted with butyl acetate (aqueous:organic, 1:2 v/v). The organic extract was evaporated as described above, and the purified MA residue was redissolved in 8 mL of deionised water. This solution was treated with 1% (w/v) activated carbon at 40 °C and 150 rpm for 15 min followed by hot vacuum filtration. The filtrate was allowed to cool gradually to room temperature, and crystallisation was initiated by seeding with a single crystal (~ 20 mg) of enantiomerically pure MA. Crystals were grown by incubating the solution in an ice bath and subsequent storage in a cold room (at 4 °C) for at least 48 h to promote crystal formation. The formed MA crystals were collected by vacuum filtration and dried at room temperature, protected from light, until a constant weight was achieved. The final purity of the isolated enantiomers was confirmed by NMR, HPLC and IR spectroscopy.

### Polymer synthesis

#### Synthesis of SAMMA (PPCM) polymer

The synthesis of SAMMA (PCCM) polymer was conducted via low-temperature, acid-catalysed polymerisation, as previously described by Zang et al. [40]. Commercially available MA was used as the reference monomer in reactions containing either 5.0 g or 0.5 g of MA. For comparative evaluation, 0.5 g of bio-produced MA (from this study) was employed under identical reaction conditions. In each reaction, MA (5.0 g or 0.5 g) was added gradually in small aliquots over a 10-min period to concentrated H_2_SO_4_ (99.5%), which had been pre-cooled to − 30 °C. The volume of H_2_SO_4_ used was adjusted based on the initial monomer quantity (2 mL per 0.5 g of MA). Throughout the addition, the reaction mixtures were continuously stirred and maintained at − 30 °C for 1 h to ensure controlled polymerisation kinetics and suppress undesirable side reactions. Subsequently, the temperature was allowed to rise gradually to room temperature, and the mixture was stirred for an additional 16 h at 500 rpm. Polymer precipitation was initiated by carefully quenching the reaction by pouring into an ice-cold water mixture (125 g ice in 50 mL distilled water), followed by vacuum filtration to collect the solid product. The crude polymer was thoroughly washed with distilled water and resuspended in 50 mL of fresh distilled water. The washing and filtration steps were repeated until the final filtrate reached a pH of 4–5, indicating effective removal of residual acid. The purified polymer was air-dried at room temperature overnight and subsequently characterised by matrix-assisted laser desorption/ionisation time-of-flight mass spectrometry (MALDI-TOF MS) as described in the Supplementary Information.

#### Synthesis of mandelide

Mandelide, the monomeric precursor for the synthesis of PM, was synthesised from commercially available (*R*)- and (*S*)-MA according to the procedure reported by Liu et al. (2007) with minor modifications [10]. MA (6.6 g, 43.4 mmol) and *p*-toluenesulfonic acid (PTSA, 0.23 g, 1.3 mmol) were introduced into a 1-L three-neck round-bottom flask fitted with a Dean–Stark apparatus and reflux condenser. The reaction was purged with nitrogen gas to establish an anaerobic environment and maintained under a continuous nitrogen flow throughout the reaction. A mixed xylenes solution (660 mL) was added via cannula, and the mixture was degassed under nitrogen for 30 min, with continuous stirring at 150 rpm at room temperature. The reaction mixture was then heated to reflux for 72 h. Upon completion, the reaction was allowed to cool to room temperature, and the precipitated product (mandelide) was collected by vacuum filtration. The crude solid product was washed sequentially with saturated aqueous sodium bicarbonate solution (3 × 200 mL) to neutralise residual acid catalyst, followed by washing with brine (3 × 200 mL). The organic phase was dried over anhydrous magnesium sulfate (MgSO4), filtered, and concentrated under reduced pressure using a rotary evaporator. The resulting crude mandelide product was recrystallised from ethyl acetate to improve purity and characterised by NMR spectroscopy.

### Analytical methods

The analytical methods employed in this study, including high-performance liquid chromatography (HPLC), liquid chromatography–tandem mass spectrometry (LC-MS/MS), nuclear magnetic resonance (NMR) spectroscopy, Fourier-transform infrared (FT-IR) spectroscopy, and matrix-assisted laser desorption/ionisation time-of-flight mass spectrometry (MALDI-TOF MS), are described in detail in the Supplementary Information.

## Results

### Engineering and screening of recombinant *E. coli* strains for MA enantiomer production

To advance the sustainability and industrial scalability of phenylalanine-derived MA enantiomer biosynthesis, we aimed to improve the direct microbial production of both (*R*)- and (*S*)-MA from renewable feedstocks in *E. coli* strains. Whilst in vivo biosynthetic routes to each MA enantiomer have previously been established in *E. coli*, reported titres, not exceeding 1 g/L, remain suboptimal for economically viable scale-up and industrial application [34, 35].

**Fig. 2:**
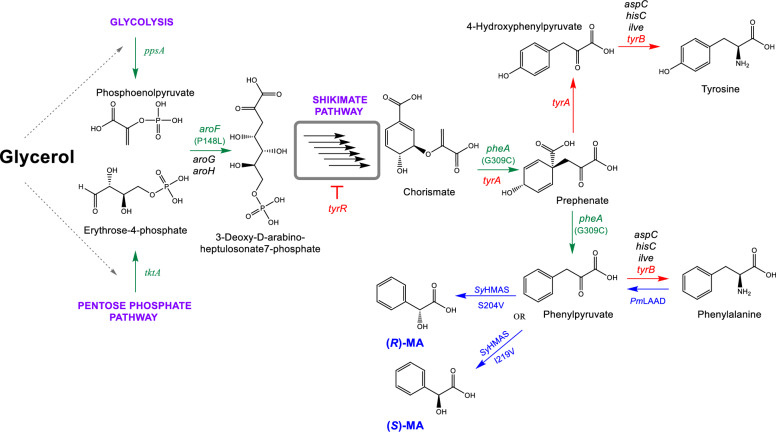
Metabolic engineering of *E. coli* DH5α strain to support the biosynthesis of MA enantiomers. The shikimate pathway uses phosphoenolpyruvate (from glycolysis) and erythrose-4-phosphate (from the pentose phosphate pathway) for the biosynthesis of aromatic amino acids. Glycerol is used as the carbon source in this study. It is converted to glyceraldehyde-3-phosphate, which feeds into both glycolysis and pentose phosphate pathway. Metabolic engineering was carried out to increase flux down the shikimate pathway towards phenylalanine. Various indicated genes encode enzymes involved in this process. Genes coloured in red were deleted from the engineered host. Genes coloured in green were overexpressed from a genome integrated construct. Mutations in *aroF* and *pheA* were introduced to relax feedback inhibition. Genes coloured in black indicate native genes that encode isozymes of genes which were deleted. Genes coloured in blue were heterologously expressed from plasmid vectors in the engineered phenylalanine overproduction strain to enable high titre production of MA. Genes encode the following enzymes: *ppsA* (phosphoenolpyruvate synthase); *tktA* (transketolase 1); *aroF*/*aroG*/*aroH* (phospho-2-dehydro-3-deoxyheptonate aldolase isozymes sensitive to tyrosine/phenylalanine/tryptophan, respectively); *tyrR* (transcriptional regulator of aromatic amino acid biosynthesis); *tyrA*/*pheA* (chorismate mutase/prephenate dehydratase isozymes specific for phenylalanine and tyrosine, respectively); *aspC*/*hisC*/*tyrB* (amino acid aminotransferases); *Pm*LAAD (*L*-amino acid deaminase from *Proteus mirabilis*); *Sy*HMAS (hydroxymandelic acid synthase from *Streptomyces yokosukanensis*

Building upon two prototype recombinant *E. coli* strains previously developed in our laboratory, capable of producing 0.80 g/L of (*R*)-MA and 0.97 g/L of (*S*)-MA [35], we sought to enhance production. This is through host strain metabolic engineering, biosynthetic pathway optimisation, improved strain stability and fermentation refinement. The initial recombinant strains, designated MA-1 and MA-2 [35], are based on the engineered *E. coli* host strain (SBC010793), harbouring a single plasmid which carries either the *Sy*HMAS(S204V) or *Sy*HMAS(I219V) gene variants for the biosynthesis of (*R*)-MA and (*S*)-MA, respectively (Table 1). SBC010793 is an engineered derivative of *E. coli* DH5α strain, constructed as a versatile phenylalanine-overproduction platform [35]. This strain incorporates rational genetic modifications including the deletion of *tyrR* (to alleviate repression of aromatic amino acid biosynthesis) and *tyrA* (to suppress tyrosine biosynthesis), thereby enhancing metabolic flux towards phenylalanine. Additionally, the strain contains a chromosomally integrated synthetic cassette at the *lacZ* locus, encoding feedback-resistant copies of *E. coli ppsA*, *aroF*, and *pheA* genes, which promote flux through the shikimate pathway towards phenylalanine biosynthesis. This rational design enables stereoselective MA biosynthesis [35] using distinct *Sy*HMAS variants directing production to the (*S*)- and (*R*)-enantiomers (Fig. 2; Table S1-S2).

Initial fermentation trials using MA-1 and MA-2 under modified conditions were conducted in an effort to improve titres; however, these trials did not yield significant improvements and exhibited significant titre variations between replicates, suggesting the persistence of upstream metabolic constraints. To address this, we introduced a targeted deletion of the *tyrB*, which encodes an aromatic amino acid aminotransferase, to reduce metabolic flux competition and enhance the intracellular availability of phenylpyruvate, a key intermediate in MA biosynthesis. This modification resulted in the construction of strain SBC015953, a derivative of SBC010793 tailored for enhanced phenylpyruvate production (Supplementary Information, Table S3 and Fig. 2). Using this host strain, two sets of enantioselective MA production strains were generated by transforming with different plasmid constructs. The first pair of strains (strains MA-3 and MA-4, Table 1) were transformed with plasmids carrying either *Sy*HMAS(S204V) for (*R*)-MA or *Sy*HMAS(I219V) for (*S*)-MA. The second pair of strains (strains MA-5 and strain MA-6, Table 1) were transformed with plasmids carrying the same *Sy*HMAS variants, along with the *Pm*LAAD gene—an engineered *L*-amino acid deaminase from *Proteus mirabilis* [41]—to convert phenylalanine back into phenylpyruvate, the precursor for MA biosynthesis (Fig. 2).

Altogether, six recombinant strains (strains MA-1 through MA-6) were systematically evaluated to investigate the impact of the additional host-strain engineering and biosynthetic pathway optimisation steps on MA biosynthesis (Table 1; Fig. 2). Comprehensive whole-genome sequencing, including plasmid sequences, was performed for all engineered strains, confirming the genomic modifications and verifying the sequence and stability of the plasmid constructs. In all the engineered strains, *ppsA* acquired an S584G mutation, located at the end of an *α*-helix adjacent to the pyruvate-binding site. This mutation may reduce enzyme activity and likely arose due to selection pressures associated with overexpression. For *tktA*, the G369C mutation was present prior to genome integration, lies at the end of an α-helix near the substrate-binding site; despite this, the construct successfully enhanced MA production, indicating that the feedback-resistant *PheA* and *AroF* genes are the primary contributors to increased flux toward phenylpyruvate. Taken together, while the *ppsA* and *tktA* mutations may reduce their respective enzyme activities, they do not appear to deleteriously compromise the effectiveness of the engineered pathway.

**Fig. 3:**
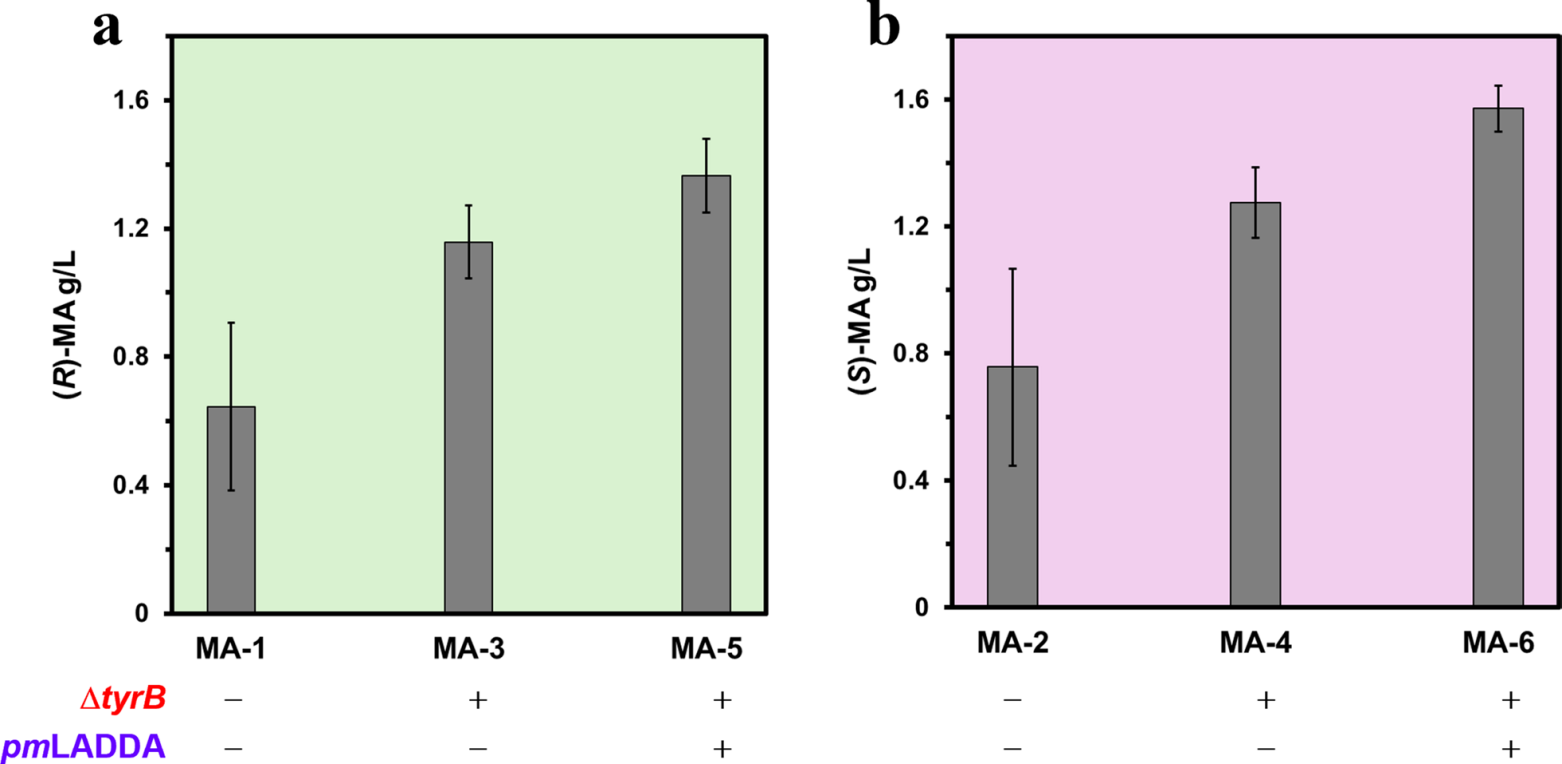
Titres of MA produced by the recombinant engineered *E. coli* strains. The indicated strains (described in Table 1) were grown in buffered TB medium (50 ml volumes) with 0.8% (w/v) glycerol, in shaking flasks at 30 °C for 72 h. The strains differ by the presence or absence of the *tyrB* deletion and the incorporation of *Pm*LAAD gene, as indicated. Data shown are means ± standard deviations calculated from replicates

The six engineered recombinant *E. coli* strains (MA-1 through MA-6) were at first screened for MA production in buffered TB medium, supplemented with 0.8% (w/v) glycerol and carbenicillin in replicate shake flasks (50 mL culture volumes) for 72 h as described in the Methods. Optical density at 600 nm (OD_600_) was recorded at the end of cultivation, and MA production was measured as described in the analytical methods section, Supplementary Information. All strains demonstrated the ability to produce MA under the tested conditions, though with varying titres (Fig. 3). For the (*R*)-MA-producing strains, titres of 0.69 ± 0.21, 1.16 ± 0.11 and 1.36 ± 0.12 g/L were observed for MA-1, MA-3 and MA-5, respectively. The (*S*)-MA-producing strains MA-2, MA-4 and MA-6, reached titres of 0.89 ± 0.26, 1.27 ± 0.11 and 1.57 ± 0.07 g/L, respectively. Based on the 8 g/L glycerol supplied, these titres correspond to overall conversion yields of 0.09 ± 0.02, 0.14 ± 0.01 and 0.17 ± 0.01 g product/g substrate for strains MA-1, MA-3 and MA-5 and yields of 0.11 ± 0.03, 0.16 ± 0.01 and 0.2 ± 0.01 g product/g substrate for strains MA-2, MA-4 and MA-6, respectively. As TB is a rich medium, these yield values represent estimates based solely on the supplemented glycerol and may underestimate contributions from additional carbon sources present in the medium. Notably, strains MA-5 and MA-6 yielded the highest titres of the (*R*)- and (*S*)-MA, respectively. These results were consistent with the intended design of the strains with both the deletion of *tyrB* and the incorporation of *pm*LAAD hypothesised to enhance metabolic flux towards the MA biosynthetic pathway by improving precursor channelling.

### Batch fermentation in TB medium

**Fig. 4:**
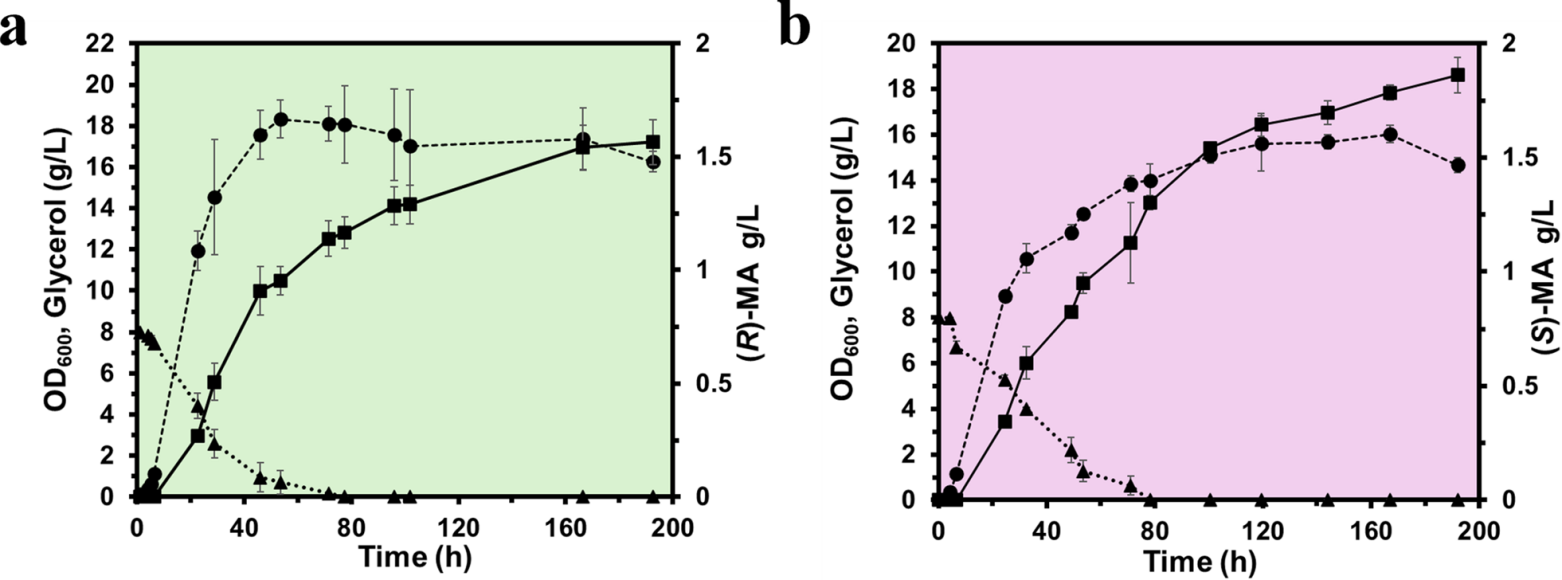
Batch fermentation in shake-flask cultures of TB medium. Strains MA-5 (**a**) and MA-6 (**b**), for the production of (*R*)-MA and (*S*)-MA, respectively, were grown in TB medium as described in the Methods. Cultures were monitored for OD_600_ (●), glycerol (▲) and MA (■). Data shown are means ± standard deviations calculated from replicates

Batch fermentations of the best performing recombinant strains, MA-5 and MA-6, were conducted in replicate 200 ml cultures in shaking flasks (1 L capacity) in TB medium supplemented with 0.8% (w/v) glycerol and carbenicillin. Fermentation runs were performed as described in the Methods section, the OD_600_ was monitored, and the consumption of glycerol and production of MA were recorded for 196 h as described in the analytical methods, Supplementary Information. The resulting titres reached 1.6 ± 0.13 g/L for (*R*)-MA and 1.86 ± 0.11 g/L for (*S*)-MA (Fig. 4), representing an approximate twofold increase compared to the previously reported titres for direct biosynthesis of MA enantiomers from glycerol [35]. Based on the 8 g/L glycerol supplied, these titres correspond to overall conversion yields of 0.20 ± 0.016 g product/g substrate for (*R*)-MA and 0.23 ± 0.014 g product/g substrate for (*S*)-MA. However, as TB is a rich medium, additional carbon sources may also contribute to product biosynthesis.

### Fed-batch fermentation in defined minimal medium

Although strains MA-5 and MA-6 demonstrated superior production in rich medium (batch fermentations in TB medium in shaking flasks, Fig. 4), subsequent fermentation experiments conducted in defined minimal medium, designed to develop conditions more relevant for scalable bioprocess development, revealed a significant limitation. Both strains exhibited markedly reduced growth and compromised viability, defined as the inability to sustain metabolic activity and cell division, resulting in growth limitation (failing to grow beyond an OD_600_ of 5.0) and failure to sustain an actively dividing population in the minimal medium under the applied fed-batch conditions used. This impaired phenotype was likely attributed to the overexpression of the *pm*LAAD, which may impose a significant metabolic burden under the conditions used by disturbing amino acid biosynthesis and nitrogen balance within the cell, thereby compromising cell growth and productivity. In contrast to rich medium such as TB, which supplies a broad range of organic nitrogen sources supporting robust growth and biosynthesis, the defined minimal medium used lacks such complexity, exacerbating the burden of heterologous *Pm*LAAD expression. These results indicate that MA-5 and MA-6 are suboptimal chassis strains for scaled or prolonged cultivation under the defined conditions tested in this study. As a result, strains MA-3 and MA-4, which lack the *Pm*LAAD gene and which also showed consistent, moderate production and robust growth (Fig. 3), were selected for the subsequent investigations in minimal medium supplemented with glycerol, under fed-batch fermentation mode.

**Fig. 5:**
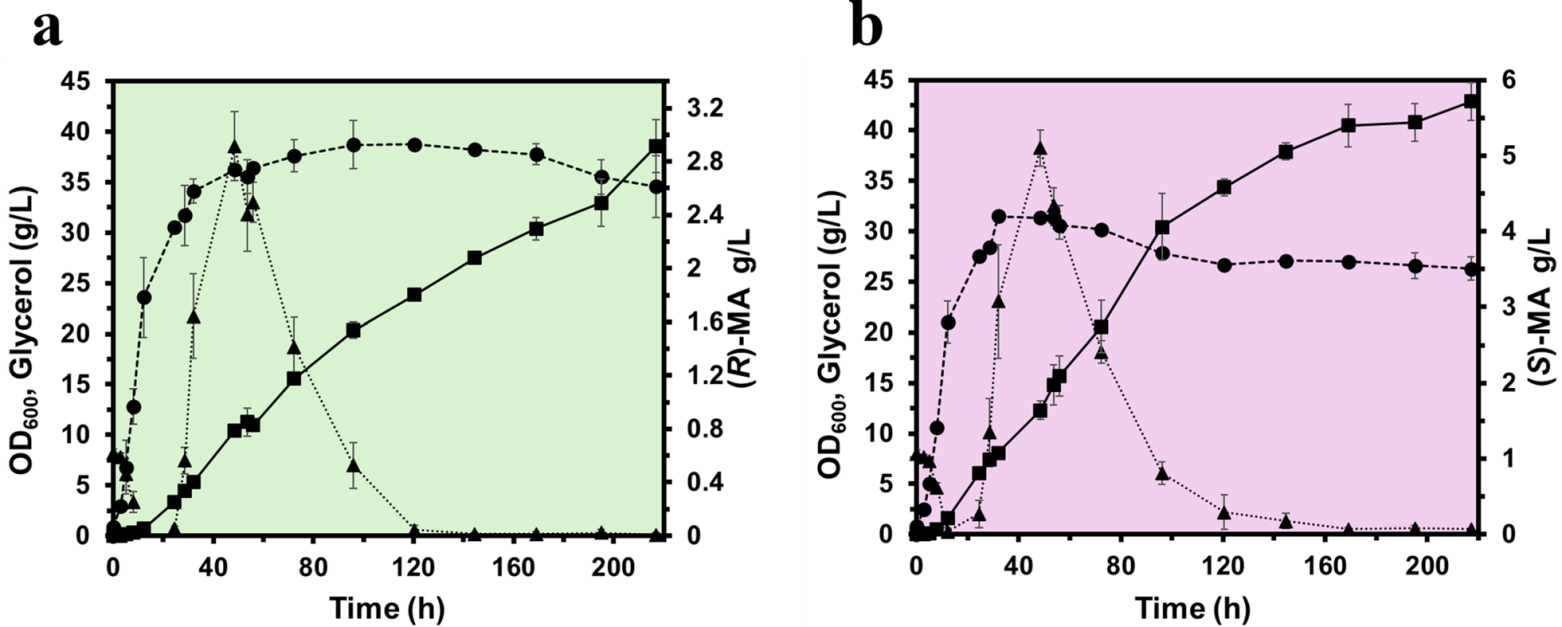
Fed-batch fermentation in bioreactor cultures of defined minimal medium. Strains MA-3 (**a**) and MA-4 (**b**) for the production of (*R*)-MA and (*S*)-MA, respectively, were grown in defined minimal medium (150 mL starting volume) with feeding in bioreactor cultures on a Sartorius AMBR250 bioreactor (Ambr® 250 modular) system. Fermentations were conducted as described in the Methods at 30°C and maintained at pH 7.0 and 30% dissolved oxygen (DO), feed rate was initially set at 0.15 mL/h and increased exponentially at a rate of 0.15 h^−1^ until reaching 5 mL/h and was maintained at this rate until the feed was administered. Cultures were monitored for OD_600_ (●), Glycerol (▲) and MA (■). Data shown are means ± standard deviations calculated from replicates

Fed-batch fermentations in defined minimal medium demonstrated that strains MA-3 and MA-4 achieved titres of 2.9 ± 0.20 g/L of (*R*)-MA [98.5% *ee*], and 5.7 ± 0.25 g/L of (*S*)-MA [93% *ee*], respectively, under our standard non-optimised bioreactor conditions used (Fig. 5). Enantiopurity was assessed and confirmed by chiral LC-MS/MS, as described in the Supplementary Information (Fig S3, Fig S4). To the best of our knowledge, these values represent the highest enantiomer titres reported to date for the direct microbial biosynthesis of both MA enantiomers from renewable carbon sources in *E. coli*. Table 2 summarises previously reported in vivo microbial MA production in *E. coli* and compares metabolic engineering strategies, cultivation conditions, titres, and enantiomeric purity with the present study.

**Table 2:** Comparison of in vivo MA production in *E. coli* reported in previous studies and the present study

Study	Metabolic strategy	Carbon source/medium	Fermentation conditions	MA enantiomer	Titre (g/L)	Purity (ee)
Sun et al., 2011 [34]	Classical *L*-Phe pathway engineering: overexpression of *hmaS* + feedback-resistant mutants of *aroF*(148L) and *pheA*(G309C); deletion of competing aminotransferases (*tyrB*, *aspC*) and branch-pathway genes (*tyrA*, *trpE*) to channel phenylpyruvate to MA for (*S*)-MA production. For (*R*)-MA, a cascade of *hmaS* → *hmo* → *dmd* was coexpressed to convert (*S*)-MA to(*R*)- MA	Glucose/defined minimal medium supplemented with *L*-Tyr, *L*-Trp, and *L*-Asp	Shake-flask batch fermentation (50 ml culture volumes); pH 7.0, 37 °C until OD_600_ of 0.4–0.6, induced with 0.1 mM IPTG, then 33 °C for 84 h	(*R*)-MA (*S*)-MA	0.88 1.02	NA 98%
Robinson et al., 2020 [35]	Biofoundry DBTL approach for production strain prototyping: engineered *L*-Phe overproducing strain with genomic cassette integrated** [***ΔlacZ::[lacI-PlacUV5-pheA*(G309C)*-ppsA*(S584G)*-aroF*(P148L)*-tktA*(G369C)] and *ΔtyrR ΔtyrA* gene knockouts. Supported (*R*)-MA and (*S*)-MA production by plasmid-expressed *Sy*HMAS (S204V) and (I219V) variants, respectively	Glycerol/SOB medium for (*S*)-MA and phosphate buffered TB medium for (*R*)-MA	Fed-batch fermentation (650 mL culture volumes in 1-L bioreactor); pH 7.0, 37 °C until OD_600_ of 2.0, induced with 0.1 mM IPTG and maintained at 37 °C, glycerol feed at 12 h intervals for 72 h	(*R*)-MA (*S*)-MA	0.8 0.97	98% 89%
Liu et al., 2025 [37]	Systematic de novo MA biosynthesis from glucose: overexpression of: *xfpK*, *tktA*, *ppsA* for improving precursor supply (PEP, E4P), and feedback-resistant mutants of *aroG*(D146N, P150L) and *pheA*(T326P); CRISPRi gene repression of competing pathways (*pykF*, *tyrR*, *trpE*) and overexpression of the HMAS3 from *Actinosynnema mirum*	Glucose/ ZYM-5052 rich medium [37]	High-density fed-batch fermentation (5-L bioreactor) with pulse feeding; pH 7.0, 37 °C, 30% DO, until OD_600_ of 30, induced with 0.2% arabinose and 1 mM IPTG, and maintained at 30 °C; pulsed feed of glucose for 86 h	Racemic (no chiral analysis)	9.58	NA
This study	Improving our previously constructed strain (Robinson et al., [35]), by *tyrB* gene deletion, to enable enhanced flux towards phenylpyruvate (rather than phenylalanine) for MA biosynthesis	Glycerol/TB medium	Shake-flask batch fermentation (250 ml volumes), pH 7.0, 30 °C until OD_600_ of 1–1.5, induced with 0.1 mM IPTG and maintained at 30 °C for 196 h	(*R*)-MA (*S*)-MA	1.6 1.86	NA NA
		Glycerol/ modified minimal medium with *L*-Tyr and additional yeast extract (4 g/L)	Feb-batch fermentation (150 mL culture volumes in Ambr 250 bioreactor); pH 7.0, 30 °C, 30% DO, until OD600 = 5.0- 6.0, induced with 0.1 mM IPTG and maintained at 30 °C, with glycerol feed for 196 h	(*R*)-MA (*S*)-MA	5.7 2.9	98.5% 93%

*aroF* and *aroG*: are genes encoding 3-deoxy-*D*-arabino-heptulosonate-7-phosphate synthases, enzymes of the shikimate/phenylalanine biosynthesis pathway; *aspC*: aminotransferase gene*;* Asp: aspartate; CRISPRi: CRISPR interference for gene repression; *dmd*: *D*-mandelate dehydrogenase gene from *R. graminis*; DBTL: design–build–test–learn pipeline; DO: dissolved oxygen; *Δ:* gene deletion; *ee*: enantiomeric excess; E4P: erythrose-4-phosphate; *fbr*: feedback-resistant allele; *hmaS:* hydroxymandelate synthase gene from *A. orientalis*; *HMAS3*: hydroxymandelate synthase gene from *Actinosynnema mirum*; *hmo*: hydroxymandelate oxidase gene from *S. coelicolor*; IPTG: isopropyl *β*-*D*-1-thiogalactopyranoside; *lac I*: gene encodes the lac repressor protein; *lacZ*: lac operon gene that encodes *B*-galactosidase; *L-*Phe: *L*-phenylalanine; MA: mandelic acid; (*R*)-MA and (*S*)-MA: are the *R*- and *S*-enantiomers of mandelic acid; OD₆₀₀: optical density at 600 nm; PEP: phosphoenolpyruvate; *pheA*: gene encoding a bifunctional chorismate mutase / prephenate dehydratase involved in *L-*Phe biosynthesis; *PlacUV5*: modified lac promoter; *ppsA*: phosphoenolpyruvate synthase gene; *pykF*: pyruvate kinase gene; SOB: super-optimal broth medium; *Sy*HMAS: hydroxymandelate synthase from *Streptomyces yokosukanensis*; TB: terrific broth; Tyr: tyrosine; *tyrA*: chorismate mutase/prephenate dehydrogenase gene; *tyrB*: aromatic amino acid aminotransferase gene; *tyrR*: transcriptional regulator of aromatic amino acid biosynthesis; Trp: tryptophan; *trpE*: anthranilate synthase gene; *tktA*: transketolase gene; *xfpK*: phosphoketolase gene

The marked improvement in titres represents a significant advancement in bioprocess development, underscoring the platform’s potential for industrial-scale production and providing a foundation for further bioprocess optimisation at larger fermentation scales.

We calculated the yield for the fed-batch fermentation relative to glycerol consumption and noted that the presence of yeast extract renders these values conservative and not directly comparable to defined-medium yields. The observed yields under the non-optimised conditions used in this study are low (0.039 ± 0.0027 g product/g substrate for (*R*)-MA and 0.076 g product/g substrate for (*S*)-MA) but remain consistent with early-stage pathway implementation that has not yet undergone expression tuning or flux optimisation.

### Recovery of MA enantiomers from fermentation broth

A couple of strategies were evaluated for the recovery of MA enantiomers from the fermentation broth, including the reactive extraction method [7, 8]. However, under the conditions tested, these approaches yielded recovery efficiencies below 37% (data not shown). The most effective recovery method was developed in this study, adapted from previously reported protocols [38, 39], and further refined as described in the Methods to enhance substantially higher recovery and purity. To clarify the basis of this improvement, the strategy centred on combining pH-controlled extraction with targeted impurity reduction and a final seeded crystallisation step. Alkaline extraction facilitated efficient partitioning of mandelate into the aqueous phase as its sodium salt, while subsequent acidification and re-extraction and treatment with activated carbon improved the removal of co-extracted impurities. The final crystallisation step, initiated by seeding with enantiomerically pure MA, enhanced product purity, collectively resulting in markedly higher recovery compared to earlier approaches.

Following fermentation, MA enantiomers were recovered from a total broth volume of 200 mL following the downstream protocol described in the Methods section. The extraction process resulted in the recovery of 0.432 g of (*R*)-MA and 0.966 g of (*S*)-MA, corresponding to recovery efficiencies of 77% and 84%, respectively (Table S4). These recovery rates demonstrate the effectiveness of the developed extraction protocol in isolating MA enantiomers from the fermentation matrix under the applied conditions.

Each purified product was characterised by ^1^H and ^13^C NMR spectroscopy in deuterium oxide (D_2_O). The observed chemical shifts matched those of authentic MA standards (Fig. S5 – S8). No extraneous peaks or unexpected signals were observed outside the expected spectral regions, indicating minimal contamination and the absence of any significant byproducts in the purified enantiomers. Additionally, signal integration corresponded perfectly with the expected proton and carbon environments, further confirming both the structural integrity and the high purity of the isolated acid enantiomers.

HPLC analysis of the purified product for both enantiomers revealed a single peak with retention times identical to the MA standard (Fig. S11, S12), confirming the chromatographic purity of the isolated enantiomers. IR spectroscopy further supported this conclusion, with spectra displaying characteristic absorption bands corresponding to the functional groups of MA (Fig. S13, S14). Collectively, these analytical results demonstrate that the developed extraction method efficiently recovered MA enantiomers from the fermentation broth, yielding products of high purity (~ 99% purity) suitable for further applications. It is noteworthy that the final purification step, involving an additional extraction in butyl acetate and crystallisation following activated charcoal treatment, was essential for markedly enhancing the purity of the MA enantiomers to 99%, as evidenced by the NMR, HPLC and IR data presented. Prior to this step, the enantiomer products exhibited slightly lower purity levels (Fig. S9-S14) compared to the final highly purified enantiomer products.

### Polymer synthesis from MA enantiomers

#### Synthesis of SAMMA polymer from bio-produced MA enantiomers

SAMMA polymer was successfully synthesised from bio-derived (*R*)- and (*S*)-MA enantiomers via an acid-catalysed condensation polymerisation reaction, as described in the Methods section. To assess the influence of monomer origin, identical reactions were carried out using both bio-produced and commercially sourced MA enantiomers for comparison. In all cases, polymerisation proceeded efficiently under identical reaction conditions, yielding pale pink polymer products. The resulting polymers were isolated in good yield and characterised by MALDI-TOF mass spectrometry (Fig. S15–S19), confirming the formation of the expected polymer backbones. Key data of these SAMMA polymers, including polymer yield and maximum observed chain length, are summarised in Table 3. The mass spectra revealed repeat unit intervals of 134 Da, consistent with the molecular weight of a MA unit minus one water molecule (152 – 18 Da), as expected for step-growth condensation. The synthesised polymers exhibited molecular weights ranging from approximately 3200 to 4000 Da (when analysed in reflection, high accuracy mode), corresponding to an average degree of polymerisation (DP) of ~ 24 – 30 monomer units per chain; however, higher molecular weights up to 6000 Da were observed when the data were analysed in linear mode. These values are in line with theoretical expectations for step-growth condensation polymerisation, which proceeds via successive monomer coupling and water elimination. Notably, polymerisation efficiency and product characteristics were consistent across both bio-derived and commercial MA enantiomers, irrespective of monomer origin or input quantity (Table 3). Despite the limited monomer yields recovered from fermentation in this study, the successful synthesis of SAMMA polymers highlights the feasibility of employing the bio-derived MA monomers directly in functional polymer synthesis.

**Table 3:** Yield and chain length of the synthesised SAMMA polymers

MA (starting material in g)	Produced polymer (g)	% Yield	Mwt (Da)*
Standard (*R*)-MA (0.5 g)	0.401	91.05	3777.96
Bio-produced (*R*)-MA (0.5 g)	0.392	88.94	4046.91
Standard (*S*)-MA (0.5 g)	0.374	84.86	3345.31
Bio-produced (*S*)-MA (0.5)	0.396	89.86	3480.47
Standard MA (5 g)	4.150	94.1	3911.91

^*^Is the maximum chain length detected by MALDI-TOF MS analysis under the conditions used

### Synthesis of the PM monomer mandelide

To further demonstrate the utility of MA in polymer chemistry, we targeted the synthesis of mandelide, the key monomeric intermediate in the production of PM. While the primary objective was to demonstrate the applicability of using bio-derived MA enantiomers obtained via fermentation in the Ambr^®^250 system, the recovered yield was insufficient at that scale to support the demanding conditions required for mandelide and subsequent PM synthesis. In particular, the ring-opening polymerisation of mandelide is a highly sensitive and technically challenging process that requires stringent control over reaction conditions—typically achievable only in specialised laboratory or industrial settings. Given these constraints, we proceeded with the synthesis of mandelide using commercially available (*R*)- and (*S*)-MA as described in the Methods section. Notably, rigorous degassing of the MA solution prior to reflux was critical for improving reaction efficiency. Optimal conversion was achieved after three days of continuous reflux. The crude mandelide product was recrystallised from ethyl acetate, affording pure mandelide (1.5 g, 26% yield for (*R*)-MA and 2.0 g, 34.4% yield for (*S*)-MA) as a pale-buff solid. Those yields are in agreement with previously reported studies producing 1.3 g of *rac*-mandelide from racemic MA [10]. Product identity was confirmed by ^1^H NMR spectroscopy, with chemical shifts consistent with literature values (Fig. S20, S21). 1H NMR (500 MHz; CDCl_3_): δ 6.14 (s, 1H), 7.28 − 7.47 (m, 5H). The results demonstrate the feasibility of progressing from MA to mandelide, thereby demonstrating a viable route towards PM synthesis.

Combined, these results illustrate the broader potential of microbially produced MA enantiomers, not only for direct polymer formation, as illustrated with SAMMA, but also as valuable intermediates for downstream polymer and copolymer synthesis.

## Discussion

This study presents a significant advancement in the microbial production of MA enantiomers, addressing long-standing challenges in their biosynthesis from renewable carbon sources. Building on our previously established pathway [35], the main focus was to enhance microbial bioproduction of both (*R*)- and (*S*)-MA from glycerol, developing an early-stage platform aligned with sustainable bioprocessing principles. Glycerol was selected as the carbon source due to its favourable metabolic characteristics and abundance in industrial waste streams, including biodiesel-derived waste streams. Moreover, its prior successful use for MA biosynthesis [32, 35] further supports this choice.

Because the primary objective of this study was to enhance metabolic flux toward phenylpyruvate (the direct substrate for *Sy*HMAS) rather than to phenylalanine, the introduction of *Sy*HMAS diverts phenylpyruvate away from phenylalanine biosynthesis, making phenylalanine quantification outside the scope of this proof-of-principle work at this stage. A phenylalanine-producing strain, which was used as the basis for our engineering system, was previously quantified for phenylalanine accumulation, reaching 2.35 g/L in rich TB medium over 24 h compared with 0.61 g/L in the wild-type strain, confirming that these genetic modifications successfully enhanced flux toward phenylalanine prior to the introduction of *Sy*HMAS (Robinson *et al*., [35]). Nonetheless, systematic assessment of phenylalanine levels in the engineered strains represents a valuable direction for future optimisation studies.

Targeted metabolic engineering of the *E*. *coli* DH5α developed in this study led to MA titres approximately fivefold higher than earlier reports for MA enantiomers, establishing a new benchmark for enantioselective microbial MA production. As summarised in Table 2, previous reports of in vivo MA production in *E. coli* either achieved titres lacking enantioselectivity or focused on enantiomerically pure MA but reached low titres, with limited attention to downstream recovery. In contrast, our study demonstrates higher titres for enantiomerically pure MA, while also integrating an efficient downstream purification process. This combination highlights the novelty and practical advantage of our approach.

The observed differences in the (*R*)- and (*S*)-MA titres reflect the distinct catalytic architectures and potential flux-controlling properties of the *Sy*HMAS variants. Robinson et al. (2020) demonstrated that wild-type *Sy*HMAS exhibits strong inherent stereochemical bias toward the *S*-enantiomer, with minimal turnover toward the *R*-enantiomer. The S204V substitution inverts this stereochemical preference, and the I219V variant retains *S*-selectivity but enhances catalytic throughput [35]. In vivo, these intrinsic kinetic differences are further modulated by strain-specific variation in expression levels, enzyme stability and other factors, collectively modulating pathway flux collectively shaping enantiomeric outputs observed in both batch (Fig. 4) and fed-batch fermentations (Fig. 5). Furthermore, the enantiomeric excess (*ee*) of the produced MA enantiomers (98.5% for (*R*)-MA and 93% for (*S*)-MA) reflects the intrinsic biocatalytic selectivity of the *Sy*HMAS variants in vivo. Deviations from perfect stereoselectivity can arise from various factors such as expression levels, intracellular substrate concentrations or competing native reactions. Future improvements could include further enzyme engineering, expression tuning, and minimising competing fluxes to further enhance stereochemical fidelity while maintaining high titres.

To enhance flux through the biosynthetic pathway and overcoming observed bottleneck, a key intervention was the deletion of *tyrB* (Fig. 2), which relieved a major bottleneck and consequently increased MA biosynthesis. Early explorations of *aspC* and *hisC* deletions (Fig. 2) were halted due to the accumulation of multiple unintended mutations that compromised pathway integrity; nevertheless, these genes, along with others, remain promising targets for future rational optimisation.

In this study, we advanced strains MA-3 and MA-4 for fed-batch evaluation, as these variants demonstrated robust growth and stable performance in defined minimal medium. Strains MA-5 and MA-6 expressing *Pm*LAAD, however, exhibited pronounced growth impairment in defined minimal medium, likely caused by a futile cycle between endogenous aromatic transaminases and *Pm*LAAD that disrupts nitrogen balance and depletes intracellular glutamine. The broad substrate range of *Pm*LAAD may further disturb intracellular amino acid pools, collectively creating substantial metabolic burden. As our aim was to establish an early-stage production platform rather than undertake extensive optimisation, these strains were not advanced further in this proof-of-principle study. Nonetheless, several strategies such as supplementation with aromatic amino acids or glutamine, adjustment of nitrogen sources, and modulating *Pm*LAAD expression via promoter or RBS engineering could mitigate these limitations and represent promising directions for future iterations of strains MA-5 and MA-6 to maintain robust growth while sustaining high MA productivity.

In both batch and fed-batch fermentation, continued MA production after glycerol depletion (Fig. 4 and Fig. 5) likely results from the accumulation of upstream intermediates in the pathway, with the heterologous synthase variants acting as a rate-limiting step. As growth slows, carbon flux is redirected toward secondary metabolite production due to reduced biomass demand and relief of precursor competition. This suggests that strategic nutrient limitation, particularly nitrogen or phosphate limitation, could be strategically applied to shift metabolism earlier toward product formation and could be explored to further enhance titres. The accumulation of glycerol between 30 and 130 h during fed-batch fermentation is attributed to the non-optimised feeding strategy employed in this study. The feed rate used was set based on a maximum growth rate of 0.15 h^−1^ determined under batch conditions. However, during the fed-batch fermentation, the culture failed to match this growth rate during the production phase and, as a result, the fixed feed rate exceeded the metabolic demand for carbon under the conditions used and resulted in extracellular glycerol accumulation. This imbalance likely had some negative impact on cell growth and may have affected MA production efficiency. Controlled-feeding strategies would be expected to minimise glycerol accumulation and enhance production performance. Such optimisation was not undertaken in this proof-of-concept study, as the primary aim was to establish the integrated microbial production platforms.

The work presented here integrates strain engineering, fermentation, purification, and application validation to provide a holistic demonstration of platform feasibility to evaluate the performance of a straightforward biosynthetic route rather than an exhaustive optimisation study. While the titres achieved here in this laboratory-scale study are substantial for early-stage development, further optimisation will be required to accelerate MA production and increase yield, and complementary approaches can be advanced at both strain and process levels for industrial translation. Future efforts will prioritise scale-up to industrially relevant conditions, where precise control over parameters, such as nutrient, feeding regime, and associated culture conditions, will be critical to maximise yield, robustness and economic viability.

Although the engineering scope is modest relative to full industrial development, the study demonstrates a clear advancement in titre, strain performance and downstream compatibility, and provides a strong foundation for subsequent rounds of systematic optimisation. The observed metabolic limitations, including precursor availability and competition with native aromatic pathways, provide clear entry points for subsequent optimisation.

To accelerate MA production and increase titres and yields, several strategies can be advanced at both the strain and process levels. At the strain level, enhancing precursor availability through targeted metabolic engineering, modulating glycerol uptake, and fine-tuning *Sy*HMAS expression and activity are promising approaches to boost pathway flux. Engineering *Pm*LAAD for improved substrate specificity, tuning its expression via promoter or RBS engineering to enhance flux toward phenylpyruvate while minimising non-target amino acids’ depletion, and targeting alternative aminotransferases involved in phenylpyruvate turnover to minimise competing fluxes are promising strategies. Adaptive laboratory evolution-type strategies could be applied to select for variants with improved growth and metabolic robustness, alleviating strain-specific growth limitations. At the process level, extended or optimised fed-batch fermentations, controlled substrate feeding strategies, and precise regulation of key physicochemical parameters are expected to further increase MA and accelerate production. Collectively, these strategies provide a roadmap to accelerate MA biosynthesis while maintaining high titres, supporting future industrial-scale implementation.

A streamlined downstream purification protocol was established in this study, enabling the recovery of high-purity MA directly from fermentation broth. This integration of upstream biosynthesis with an effective purification strategy is essential for the economic feasibility of microbial manufacturing processes. The recovery efficiency of the MA enantiomers from fermentation broth could be further improved by optimising pH-controlled extraction, employing two-phase partitioning systems to reduce the number of purification steps, and exploring ion-exchange chromatography conditions tailored specifically for MA. Additionally, reducing by-product formation through metabolic engineering may simplify purification and improve overall recovery efficiency.

The compatibility of the bio-produced MA enantiomers with established downstream chemistries was demonstrated through polymerisation studies. While the polymerisation itself is not presented as a chemical innovation, the successful synthesis of SAMMA, a pharmaceutically relevant polymer with antiviral and contraceptive properties, from the bio-produced MA enantiomers demonstrates and validates that the microbial products meet the purity and structural requirements for materials applications. While mandelide synthesis relied on commercial MA due mainly to the limited bio-MA recovered from early-stage benchtop fermentations, the demonstration underscores the broader synthetic versatility of MA enantiomers. The use of commercial MA for mandelide synthesis was necessitated by the limited quantity of bio-derived MA recovered; however, strategies described above, including optimised fermentation strategies, substrate feeding, and pathway engineering, as discussed earlier would enable the production of sufficient bio-MA to enable bio-based mandelide synthesis, further validating the platform’s potential for integrated production of the PM functional material.

This proof-of-concept study employed a plasmid-based expression system to allow rapid evaluation of the pathway and to establish the integrated production–purification workflow. For industrial implementation, chromosomal integration will be essential to eliminate antibiotic dependence and ensure long-term genetic stability, and new surveillance of technologies and optimisations in each component of the process will be essential for industrial translation. These future developments lie beyond the scope of the present work but represent natural extensions of the platform described here and provide a clear path towards an industrially robust MA biomanufacturing platform.

In summary, this study establishes a high-titre microbial platform for sustainable MA enantiomer production from glycerol, supported by efficient downstream recovery and functionally validated through successful synthesis of functional materials. By bridging metabolic engineering with application-driven materials chemistry, it demonstrates the potential of microbial biomanufacturing to deliver scalable and environmentally responsible routes to chiral building blocks and next-generation biopolymers. Future efforts that optimise fermentation strategies, accelerate production, and increase bio-MA yields will further strengthen the platform’s industrial and functional relevance.

## Conclusions

This study establishes a forward-looking biosynthetic platform for the production of MA enantiomers, demonstrating the strategic integration of microbial engineering, downstream process design, and application-oriented validation. It achieves a marked increase in production titres and demonstrates the direct translation of pathway optimisation into functionally relevant, high-purity monomers for advanced material synthesis. By extending the functionality of biosynthesised MA towards polymer synthesis, this work validates both the chemical fidelity and applicability of the microbially derived product, whilst opening new avenues in biomanufacturing-enabled materials chemistry. The platform’s modularity and compatibility with downstream chemical transformations highlight its versatility for adaptation to a diverse array of bio-based compounds and applications.

Looking ahead, scaling production through optimisation of fed-batch or continuous fermentation will be critical to unlock the system’s full industrial potential. Furthermore, integrating this platform with cost-effective, renewable feedstocks or carbon-rich industrial waste streams presents further opportunities to enhance environmental sustainability and economic competitiveness. Expanding the portfolio of MA-derived materials will consolidate the platform’s strategic relevance in sustainable pharmaceuticals and bio-based materials development.

Collectively, this work lays a robust foundation for advancing biomanufacturing-enabled polymer chemistry, and supports the vision of a circular, bio-based economy. The integration of microbial biosynthesis with functional materials development exemplifies a pivotal step towards renewable, sustainable manufacturing paradigms, positioning MA not only as a high-value chiral intermediate, but also as a key component in the development of renewable, next-generation bio-based materials.

## Supplementary Information

Supplementary file 1.

## Data Availability

Data are provided within the manuscript or supplementary information file.
