# Supplementary material for: Enhanced bioproduction and processing of mandelic acid enantiomers: towards a sustainable platform for high-value pharmaceutical and polymer applications
Source: Biotechnol Biofuels Bioprod. 2025 Dec 17;19:4. doi: 10.1186/s13068-025-02727-1 (PMC12822336; doi:10.1186/s13068-025-02727-1)
Supplement: Supplementary file 1 [file 13068_2025_2727_MOESM1_ESM.docx]

**Supplementary Information**

**Enhanced Bioproduction and Processing of Mandelic Acid Enantiomers: Towards a Sustainable Platform for High-value Pharmaceutical and Polymer Applications**

Hanan Latif Messiha^1,2^, Alec Banner^1,2^, Mohamed Amer^1,2^, Christopher James Robinson^1,2^, Aula Alwattar^2^, Viranga Tilakaratna, Rosalind Le Feuvre^1,2^ and Nigel Shaun Scrutton^1,2^**^*^**

^1^ Future Biomanufacturing Research Hub (Future BRH), Manchester Institute of Biotechnology (MIB), 131 Princess Street, M1 7DN, Manchester, UK

^2^ Department of Chemistry, School of Natural Sciences, Faculty of Science and Engineering, University of Manchester, Oxford Road, M1 9PL, Manchester, UK

**^*^**Correspondence: [nigel.scrutton@manchester.ac.uk](mailto:nigel.scrutton@manchester.ac.uk)

**Table of Contents**

**Page**

**Experimental Details** 3

*Construction of gene expression plasmids for E. coli* 3

*Construction of gene knockout plasmids* 3

*Construction of engineered E. coli strains* 4

*Preparation of chemically competent cells* 5

*Transformation Protocol* 5

**Table S1** List of plasmids used in the study 6

**Table S2** List of primers used in the study 7

**Table S3** List of strains used in the study 8

**Analytical Methods** 9

*High performance liquid chromatography (HPLC)* 9

*Liquid Chromatography- Tandem Mass Spectrometry (LC-MS/MS)* 9

*Nuclear magnetic resonance (NMR) spectroscopy* 10

*Fourier Transform Infra-red (FT-IR) spectroscopy* 10

*Matrix-assisted laser desorption/ionization time-of-flight mass spectrometry*

*(MALDI-TOF MS)* 10

**Figure S1** Representative HPLC chromatograms for the analysis of MA and MA standard curve 11

**Figure S2** Representative HPLC chromatograms for the analysis of glycerol and glycerol

standard curve 12

**Figure S3** Representative LC-MS/MS chromatograms for the chiral analysis of MA 13

**Figure S4** Standard curves of MA enantiomers 14

**Table S4** Quantitative recovery and purification of MA isomers from

fermentation broth 15

**Figure S5** ^1^H NMR spectra of bioproduced (*R*)-MA and standard (*R*)-MA 16

**Figure S6** ^13^CNMR spectra of bioproduced (*R*)-MA and standard (*R*)-MA 17

**Figure S7** ^1^H NMR spectra of bioproduced (*S*)-MA and standard (*S*)-MA 18

**Figure S8** ^13^C NMR spectra of bioproduced (*S*)-MA and standard (*S*)-MA 19

**Figure S9** ^1^H NMR spectra of crude product of (*R*)- and (*S*)-MA 20

**Figure S10** ^13^C NMR spectra of crude product of (*R*)- and (*S*)-MA 21

**Figure S11** HPLC analysis recording the chromatogram of the purified

fermentation-derived (*R*)-MA 22

**Figure S12** HPLC analysis recording the chromatogram of the purified

fermentation-derived (*S*)-MA 22

**Figure S13** IR spectra of the purified fermentation-derived (*R*)-MA 23

**Figure S14** IR spectra of the purified fermentation-derived (*S*)-MA 24

**Figure S15** Mass Spectrum of SAMMA synthesised from standard (*R*)-MA.

(0.5 g starting material) 25

**Figure S16** Mass Spectrum of SAMMA synthesised from the bioproduced (*R*)-MA

(0.5 g starting material) 26

**Figure S17** Mass Spectrum of SAMMA synthesised from standard (*S*)-MA

(0.5 g starting material) 27

**Figure S18** Mass Spectrum of SAMMA synthesised from the bioproduced (*S*)-MA

(0.5 g starting material) 28

**Figure S19** Mass Spectrum of SAMMA synthesised from standard MA

(5 g starting material) 29

**Figure S20** ^1^H NMR spectrum of (*R*), (*R*)-mandelide in CDCl_3_ 30

**Figure S21** ^1^H NMR spectrum of (*S*), (*S*)-mandelide in CDCl_3_ 31

**References** 32

**Experimental Details:**

**Construction of gene expression plasmids for *E. coli***

Variants of 4-hydroxymandelate synthase (HMAS) from *Streptomyces yokosukanensis* (UniProt ID: A0A101NRJ5) were screened previously (1) to identify the mutants *Sy*HMAS(S204V) and *Sy*HMAS(I219V) which preferentially produce (*R*)- and (*S*)-enantiomers of MA, respectively. The genes encoding these variants were cloned into the pBbB1a vector to generate plasmids SBC010825 and SBC010826 (Table S1). A 1503 bp DNA construct encoding an *L*-amino acid deaminase (*Pm*LAAD) from *Proteus mirabilis* (UniProt ID: B4EZ74), incorporating D165K/F263M/L336M mutations to improve catalytic activity (2), was designed with an upstream ribosome binding site using the PartsGenie software (3), and synthesised (plasmid SBC015936). The *Pm*LAAD gene was cloned into plasmids SBC010825 and SBC010826 directly downstream of the *Sy*HMAS stop codon. The plasmids were linearized by PCR using the primers pBb_Fw and HMAS_Rv (Table S2), while the *Pm*LAAD construct was PCR-amplified with primers HMAS_LAAD_Rv and LAAD_pBb_Rv (Table S2), incorporating 15-bp overlapping sequences for In-Fusion Snap Assembly (Takara Bio). This cloning strategy yielded plasmids SBC015937 and SBC015938 (Table S1), which were verified by whole-plasmid sequencing.

**Construction of gene knockout plasmids**

A 1284 bp *tyrB* knockout construct, comprising a *tyrB*-targeting CRISPR array targeting *tyrB* and a donor DNA sequence for dsDNA break repair, was designed and synthesised. The donor DNA included a short 'cargo' sequence flanked by 500 bp homology arms corresponding to regions immediately upstream (including the start codon) and downstream (including the final 18 nucleotides and stop codon) of the *tyrB* gene in the *E. coli* genome. This design ensures the insertion of a short, transcribed scar peptide at the D*tyrB* locus, intended to minimise polar effects on adjacent genes. The pTargetF plasmid (Table S1, (4)); was linearised by PCR using primers pTargetopen-F and pTargetopen-R (Table S2). The *tyrB* knockout construct was PCR amplified with the primers pTF-tyrB_Fw and pTF-tyrB_Rv (Table S2), introducing 15-bp complementary overhangs for In-Fusion Snap Assembly (Takara Bio). The resulting plasmid SBC015994 (pTF-del(*tyrB*)), was generated (Table S1) and is compatible with our established CRISPR genome engineering toolkit (5).

**Construction of engineered *E. coli* strains**

The engineered *E. coli* strains SBC007570 and SBC010793 (Table S3), derived from *E. coli* DH5α, are tyrosine autotrophs with enhanced phenylalanine production and were previously constructed in our laboratory (1). These strains carry *tyrR* and *tyrA* gene knockouts, whilst SBC010793 additionally harbours a four-gene construct integrated at the *lacZ* locus. This construct includes *ppsA*, feedback-resistant *pheA*(G309C) and *aroF*(P148L) genes, and the *tktA* gene (carrying an unintended G369C mutation). For this study, an additional gene knockout of *tyrB* (encoding aromatic amino acid aminotransferase) was introduced into strains SBC007570 and SBC010793, yielding strains SBC015996 and SBC015953 (Table S3).

Gene knockouts were performed using our CRISPR toolkit (5). Knockout constructs were designed, synthesised, and cloned into the pTargetF plasmid (Table S1) to generate pTF-del(*gene*) constructs, as described previously. Cells were co-transformed with pSIMcpf1 (Table S1**)** and the appropriate pTF-del(*gene*) plasmid, to initiate the CRISPR-mediated genome cleavage and homology-directed repair. Following genome editing, cells were cured of both plasmids, then subjected to whole genome sequencing to confirm incorporation of the desired genetic modifications. Genomic DNA (gDNA) was extracted using the Monarch gDNA Extraction Kit (New England Biolabs), following the Gram- negative 'no lysozyme' protocol. gDNA concentrations were quantified using a Qubit fluorometer with the dsDNA Broad-Range Assay Kit (Thermo Fisher Scientific), and purity was assessed using a NanoDrop spectrophotometer (Thermo Fisher Scientific).

**Preparation of chemically competent cells**

Chemically competent *E. coli* cells were prepared for the tested strains (SBC010793 and SBC015953) using the standard calcium chloride method. Briefly, single colonies of each strain were inoculated into 5 mL of Luria-Bertani (LB) medium and incubated overnight at 37 °C with shaking (180 rpm). The overnight cultures were diluted 1:100 into 50 mL of fresh LB medium and grown at 37 °C until an optical density at 600 nm (OD₆₀₀) of 0.4–0.6 was reached. Cultures were chilled on ice for 15 min, harvested by centrifugation at 4,000 × *g* for 10 min at 4 °C, and suspended in 20 mL of ice-cold 0.1 M CaCl_2_ and incubated on ice for 30 min. Cells were pelleted again and resuspended in 2 mL ice-cold 0.1 M CaCl_2_ containing 20% (v/v) glycerol. Aliquots (50–100 μL) were flash frozen in liquid nitrogen and stored at –80 °C until use.

**Transformation Protocol**

For transformation, 50 μL of chemically prepared competent cells of the engineered strains (SBC010793 or SBC015953) prepared as explained in the supporting information were mixed with 1–5 μL (10–100 ng) of the desired plasmid DNA and incubated on ice for 30 min. Cells were then heat-shocked at 42 °C for 45 s, immediately placed on ice for 2 min, and recovered in 950 μL of SOC medium at 37 °C with agitation (180 rpm) for 1 h to allow recovery. Following recovery, 100–200 μL of the transformation mixture was plated on LB agar medium supplemented with carbenicillin (100µg/mL) and incubated overnight at 30 °C. Positive transformants were cultured in LB broth supplemented with carbenicillin at 30 °C and shaking at 180 rpm. Transformed strains were sequenced to confirm the presence of the intended genome modifications and to verify the introduced plasmids sequence.

**Table S1 List of plasmids used in the study**

| **Plasmid ID** | **Description** | **Size (bp)** | **Replication origin** | **Resistance** | **Induction** | **Notes** | **Source** |
| --- | --- | --- | --- | --- | --- | --- | --- |
| - | pTargetF (pTF), Addgene #62226 | 2,117 | ColE1 | Streptomycin | Constitutive | Targeted CRISPR gene knock-outs | (6) |
| SBC012916 | pSIMcpf1, Addgene #153034 | 12,415 | SC101 | Hygromycin | Arabinose/T. | Dual CRISPR Cas12a (Cpf1) expression and λ-RED recombination. | (5) |
| SBC015994 | pTF-del(tyrB) | 3,270 | ColE1 | Streptomycin | Constitutive | Targeted CRISPR knockout of tyrB gene. | this study |
| SBC008376 | pBbS5k-*pheA(G309C)-ppsA-aroF(P148L)-tktA(G369C)* | 11,936 | SC101 | Kanamycin | IPTG | Enhanced phenylalanine production (inactive *tktA* gene) | (1) |
| SBC010825 | pBbB1a-*Sy*HMAS(S204V) | 5,252 | BBR1 | Ampicillin | IPTG | (*R*)-Mandelic acid production | (1) |
| SBC010826 | pBbB1a-*Sy*HMAS(I219V) | 5,252 | BBR1 | Ampicillin | IPTG | (*S*)-Mandelic acid production | (1) |
| SBC015936 | pBbA1a-*Pm*LAAD(D165K/F263M/L336M) | 5,077 | p15A | Ampicillin | IPTG | *L*-amino acid deaminase expression construct | this study |
| SBC015937 | pBbB1a-*Sy*HMAS(S204V)-*Pm*LAAD(D165K,F263M,L336M) | 6,755 | BBR1 | Ampicillin | IPTG | Enhanced (*R*)-mandelic acid production | this study |
| SBC015938 | pBbB1a-*Sy*HMAS(I219V)-*Pm*LAAD(D165K,F263M,L336M) | 6,755 | BBR1 | Ampicillin | IPTG | Enhanced (*S*)-mandelic acid production | this study |

**Table S2 List of primers used in the study**

| **Primer name** | **Sequence (5′ to 3′)** | **Description** |
| --- | --- | --- |
| pBb_Fw | GGATCCAAACTCGAGTAAGGATCTCCAG | Vector PCR for InFusion |
| HMAS_Rv | TTAGTGTTCTCTTCCGGTGCCG | Vector PCR for InFusion |
| HMAS_LAAD_Fw | GGAAGAGAACACTAAGACAGGCCTTGCCGAACG | Insert PCR for InFusion |
| LAAD_pBb_Rv | CTCGAGTTTGGATCCTTATTTCTTAAAGCGATCCAGTGAAAACGG | Insert PCR for InFusion |
| lacI_cPCR_Fw | TAATGCAGCTGGCACGACAGG | Colony PCR for *Pm*LAAD cloning |
| BBR1_cPCR_Rv | GCGAGCAGCAACTACCGACC | Colony PCR for *Pm*LAAD cloning |
| pTargetopen-F | TGAATTCTCTAGAGTCGACCTGC | Vector PCR for InFusion |
| pTargetopen-R | ACTAGTATTATACCTAGGACTGAGC | Vector PCR for InFusion |
| pTF-tyrB_Fw | AGGTATAATACTAGTAATTTCTACTCTTGTAGATATGACCTGTTGG | Insert PCR for InFusion |
| pTF-tyrB_Rv | ACTCTAGAGAATTCAAGACTTTATGTATATTTGCACG | Insert PCR for InFusion |
| pTF_seq_Fw | GCATCTGTGCGGTATTTCACACC | Sanger sequencing of pTF plasmids |
| pTF_seq_Rv | CTGTTGCAAATAGTCGGTGG | Sanger sequencing of pTF plasmids |
| t*yrB*_chk_Fw | CCTGGGTAAGCGAACGTGATACC | Colony PCR for *tyrB* knockout |
| *tyrB*_chk_Rv | CAAGTTCTATTGCAAGTAAAGTTTTTCACGC | Colony PCR for *tyrB* knockout |

**Table S3 List of strains used in the study**

| **Strain ID** | **Name** | **Genotype** | **Source** | **Description** |
| --- | --- | --- | --- | --- |
| SBC001453 | DH5α | K-12 F– λ– *fhuA2 Δ(argF-lacZ)U169 phoA glnV44 Φ80 Δ(lacZ)M15 gyrA96 recA1 relA1 endA1 thi-1 hsdR17* | NEB (C2988) | Parental strain of mutant strains below |
| SBC007570 | DH5α Δ*tyrR* Δ*tyrA* | DH5α *ΔtyrR ΔtyrA* | (1) | Phenylalanine overproduction |
| SBC010793 | DH5α Δ*tyrR* Δ*tyrA* lacZ::8376 | DH5α *ΔtyrR ΔtyrA ΔlacZ::[lacI-PlacUV5-pheA(G309C)-ppsA(S584G)-aroF(P148L)-tktA(G369C)]* | (1) | Phenylalanine overproduction |
| SBC015953 | DH5α Δ*tyrR* Δ*tyrA* Δ*tyrB* lacZ::8376 | DH5α *ΔtyrR ΔtyrA ΔtyrB ΔlacZ::[lacI-PlacUV5-pheA(G309C)-ppsA(S584G)-aroF(P148L)-tktA(G369C)]* | this study | Phenylpyruvate overproduction |
| SBC015996 | DH5α Δ*tyrR* Δ*tyrA* Δ*tyrB* | DH5α *ΔtyrR ΔtyrA ΔtyrB* | this study | Phenylpyruvate overproduction |

Underlined: mutations were identified by sequencing the whole genome of the strains and the plasmids – the results and data in this study were executed using these strains. The *tktA(G369C)* mutation was already known to be present in plasmid SBC008376 and the SBC010793 strain which was employed in our earlier study (1). The *ppsA(S584G)* mutation was discovered following genome sequencing of strain SBC015953 and its parent strain SBC010793 (it was not present in plasmid SBC08376). It is not clear whether the (S584G) mutation compromises *ppsA* function.

**Analytical Methods**

**High performance liquid chromatography (HPLC)**

Quantitative analysis of MA and glycerol was performed using an Agilent 1260 Infinity HPLC system equipped with an autosampler (1260 ALS), a column heater (TCC SL), DAD and a refractive index detector (1260 RID). Measurements were performed on an Agilent Hi‐Plex H column (300 × 7.7 mm, 8 µm) with 2.5 mM H_2_SO_4_ as the mobile phase at 60 °C, with a flow rate of 0.7 mL min-^1^ for 40 min under isocratic conditions. Filtered samples (10 µL injection volume) were analysed. MA was quantified by monitoring its UV absorbance at 210 nm, while glycerol concentrations were determined using refractive index (RI) detection, with quantification based on integrated peak areas. Calibration curves generated from authentic standards of MA and glycerol processed under identical conditions were used for quantification. Representative chromatograms and standard curves for MA and glycerol are shown in Figures S1 and S2.

**Chiral Liquid Chromatography- Tandem Mass Spectrometry (LC-MS/MS) Analysis of MA**

Chiral analysis of MA enantiomers was conducted by LC-MS/MS using an Agilent 1260 Inffinity II series liquid chromatography system coupled to an agilent 6130 quadrupole mass spectrometer equipped with an electrospray ionization source (ESI). Separation was performed on an Astec CHIROBIOTIC T column (250 mm × 4.6 mm, 5 μm) maintained at 25 °C, with a flow rate 0.5 mL/min. Optimal enantiometric separation was achieved using a binary mobile phase consisting of A (H_2_O with 0.05% formic acid) and B (Methanol with 0.05% formic acid). The gradient elution program was as follows: 0−12 min, 99% A; 12−13 min, 98−2% A; 13−17 min, hold at 2% A; 17−18 min, 2−90% A; 18−25 min, hold at 90% A, 25−26 min, 90−99% A, 26−40 min, hold at 99% A. The injection volume was 0.5 μl. MA enantiomers were detected in negative electrospray ionisation (ESI−) mode at *m/z* 151.02 (eV). Representative chromatograms are provided in Figure S3, and standard curves are shown in Figure S4.

**Nuclear magnetic resonance (NMR) spectroscopy**

Structural and purity analyses of MA and mandelide were performed either at the NMR facility, Manchester Institute of Biotechnology (MIB), or at the Chemistry Building NMR facility, University of Manchester, UK. Proton (^1^H) and carbon (^13^C) NMR spectra were acquired on either a 400 MHz Bruker spectrometer or a 500 MHz Bruker spectrometer (in the case of mandelide). Coupling constants (J) were recorded in hertz (Hz) and chemical shifts (δ) are reported in parts per million (ppm). Samples were dissolved in deuterated solvents appropriate for the analyte and referenced to the residual solvent peak.

**Fourier Transform Infra-red (FT-IR) spectroscopy**

IR spectra for MA enantiomers were recorded on a Bruker ALPHA II FT-IR spectrometer. Solid-state samples were analysed using attenuated total reflectance (ATR) mode. Spectral data were recorded over a range of 4000–400 cm⁻¹.

**Matrix-assisted laser desorption/ionization time-of-flight mass spectrometry (MALDI-TOF MS)**

Mass spectrometric analysis of SAMMA polymer was performed at the MIB Mass Spectrometry Facility using a Bruker Rapiflex TissueTyper MALDI-TOF instrument. Spectra were acquired in positive reflection mode (mass-to charge (m/z) 400-5000) or in positive linear ionisation mode (m/z 1000-10000). Laser power was manually adjusted to optimise ionisation efficiency and spectral resolution. Each spectrum was generated by averaging 10,000 laser shots collected across 50 randomly selected positions within the sample spot. Samples were prepared using the standard dry-droplet method, employing *α*-cyano-4-hydroxycinnamic acid (CHCA) as the matrix (10 mg/mL in methanol). A 1uL aliquot of the sample/matrix mixture was applied onto a ground steel MALDI target plate and air-dried prior to analysis.

**a**

**b**

**
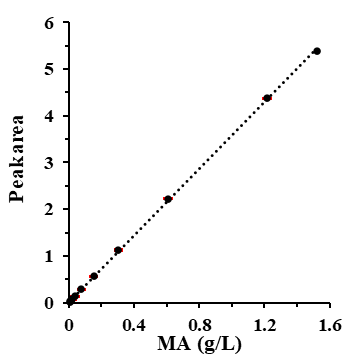
**

**c**

**Figure S1 Representative HPLC chromatograms for the analysis of MA and MA standard curve.** (**a**) Standard MA solution (0.3 g/L); (**b**) Fermentation medium from fed-batch fermentation (1:5 dilution) of (*S*)-MA product; overlay of chromatograms of (a) and (b) is presented to show retention times of the standard and sample are identical; (**c**) Calibration curve for MA. Analysis was performed as described in the Analytical Methods section, using an Agilent Hi‐Plex H column (300 × 7.7 mm, 8 µm) with 2.5 mM H_2_SO_4_ as the mobile phase, at 60 °C and a flow rate of 0.7 mL/min for 40 min. The injection volume was 10 µL. MA was quantified based on peak area, monitored by UV absorbance at 210 nm (retention time of 18.9 min).

**a**

**b**

**
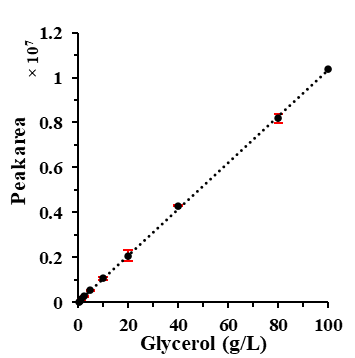
**

**c**

**Figure S2 Representative HPLC chromatograms for the analysis of glycerol and glycerol standard curve.** (**a**) Standard glycerol solution (0.3 g/L); (**b**) Fermentation medium from fed-batch fermentation (1:5 dilution); (**c**) Calibration curve for glycerol. Analysis was performed as described in the Analytical Methods section using an Agilent Hi‐Plex H column (300 × 7.7 mm, 8 µm) with 2.5 mM H_2_SO_4_ as the mobile phase, at 60 °C and a flow rate of 0.7 mL/min for 40 min. the injection volume was 10 µL. Glycerol was quantified based on integrated peak areas using refractive index (RI) detection (retention time of 13.6 min).


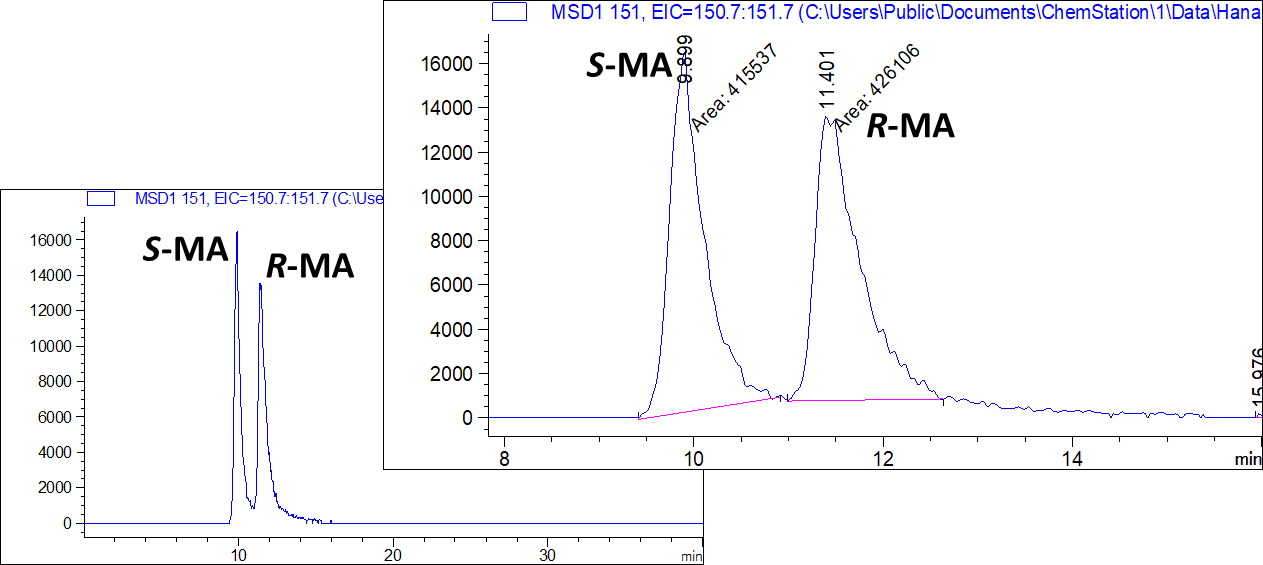


**a**

**
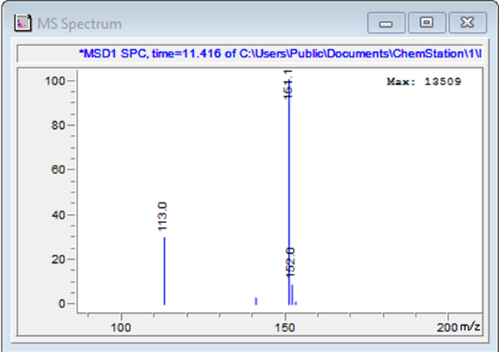
b**

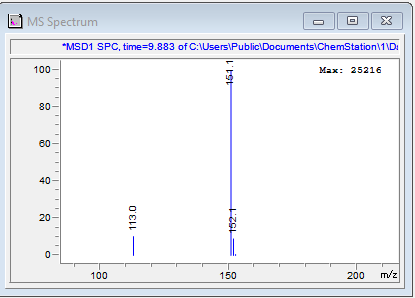


**c**

**Figure S3** **Representative LC-MS/MS chromatograms for the chiral analysis of MA,** performed as described in the Analytical Methods section**.** (**a**) Standard solutions of (*S*)-MA and (*R)*-MA (0.19 g/L each); (**b**) (*S*)-MA product produced by fed-batch fermentation in this study diluted 1:10; (**c**) (*R*)-MA product produced by fed-batch fermentation in this study diluted 1:10. Separation was achieved using an Astec CHIROBIOTIC T column (250 mm × 4.6 mm, 5 μm) at 25 °C, with a flow rate 0.5 mL/min. Mobile phase A consisted of H2O with 0.05% formic acid and mobile phase B was methanol with 0.05% formic acid. The gradient elution program was as follows: 0−12 min, 99% A; 12−13 min, 98−2% A; 13−17 min, hold at 2% A; 17−18 min, 2−90% A; 18−25 min, hold at 90% A, 25−26 min, 90−99% A, 26−40 min, hold at 99% A. The injection volume was 0.5 μL. MA enantiomers were monitored in negative electrospray ionisation (ESI−) mode.

**
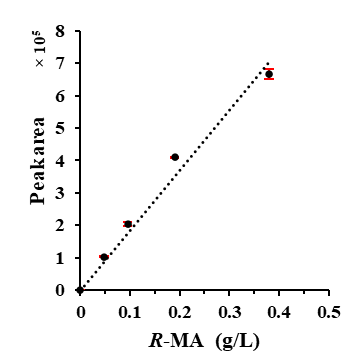
**

**
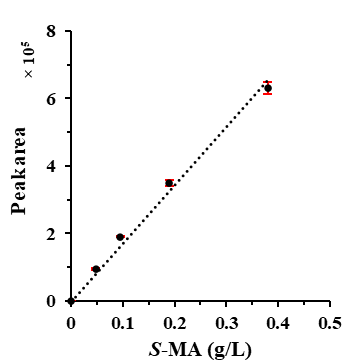
**

**Figure S4 Standard curves of MA enantiomers.** Chiral analysis of MA enantiomers was performed by LC-MS/MS, as described in the Analytical Methods section. Conditions: An Astec CHIROBIOTIC T column (250 mm X 4.6 mm, 5 μm) was used at 25 °C, with a flow rate 0.5 mL/min. Mobile phase A was H2O with 0.05% formic acid and mobile phase B was methanol with 0.05% formic acid. The gradient elution program was as follows: 0−12 min, 99% A; 12−13 min, 98−2% A; 13−17 min, hold at 2% A; 17−18 min, 2−90% A; 18−25 min, hold at 90% A, 25−26 min, 90−99% A, 26−40 min, hold at 99% A. The injection volume was 0.5 μL. MA enantiomers were monitored in ESI− mode.

**Table S4 Quantitative recovery and purification of MA isomers from fermentation broth.**

| **Step** | **(*R*)-MA** | **Loss (%)** | **(*S*)-MA** | **Loss (%)** |
| --- | --- | --- | --- | --- |
| Fermentation product | 0.561 g | N/A | 1.149 g | **N/A** |
| After alkalinization, extraction with butyl acetate (2:1) and activated charcoal treatment | 0.508 g | 9.30 % | 1.087 g | 5.37 % |
| After acidification and extraction with butyl acetate (1:2) | 0.033 g | 5.84 % | 0.031 g | 2.73 % |
| Cumulative loss so far | − | 15.137 % | − | 8.095 % |
| Purified isomer quantity (after charcoal treatment and crystallisation) | 0.4321 g | 22.97 %* | 0.9666 g | 15.91%* |
| % recovery of pure isomer | **77.03%** | | **84.094 %** | |

Loss indicates the percentage of material lost during each individual purification step. Cumulative loss represents the sum of material lost in all preceding steps up to that point, allowing tracking of overall losses prior to the final crystallisation. The total percentage loss for the complete purification process is highlighted with an asterisk (*).

The MA isomer products (each was derived from 200 mL fermentation broth volumes) were extracted and crystallised following the downstream processing protocol detailed in the Methods section. Samples were collected at each stage of the process, and quantified by HPLC analysis, as described in the Analytical Methods section, and accounting for the sample dilutions. Final yields of purified enantiomers were determined by weighing the dried isolated products.

**a**

**b**

**Figure S5 ^1^H NMR spectra of purified bioproduced (*R*)-MA (a) and standard (*R*)-MA (b).** Samples were dissolved in D_2_O (30 mg/mL).

**a**

**b**

**Figure S6 ^13^CNMR spectra of purified bioproduced (*R*)-MA (a) and standard (*R*)-MA (b).** Samples were dissolved in D_2_O (30 mg/mL).

**a**

**b**

**Figure S7 ^1^H NMR spectra of purified bioproduced (*S*)-MA (a) and standard (*S*)-MA (b).** Samples were dissolved in D_2_O (30 mg/mL).

**
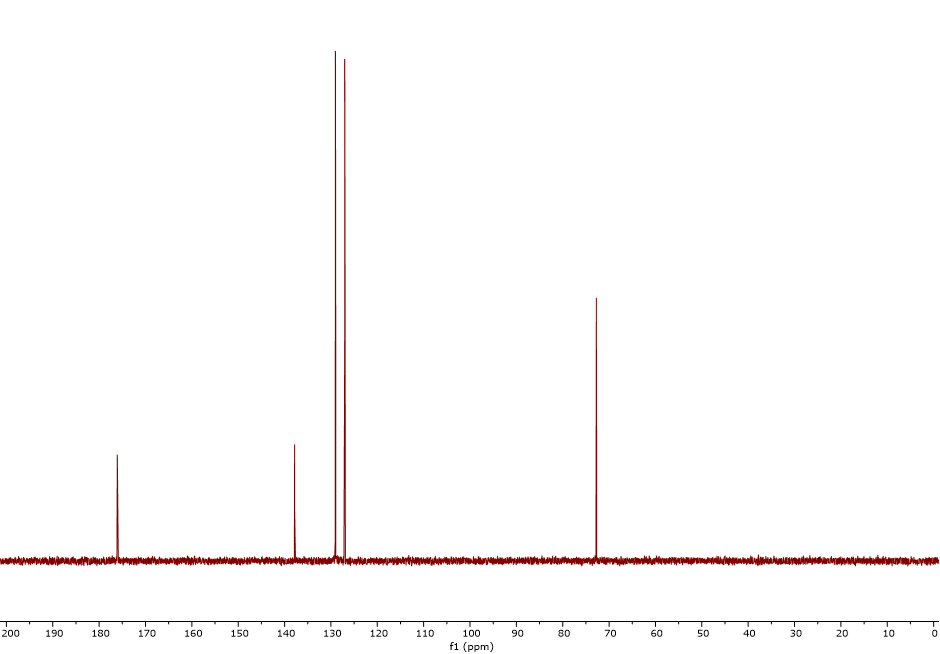
a**

**
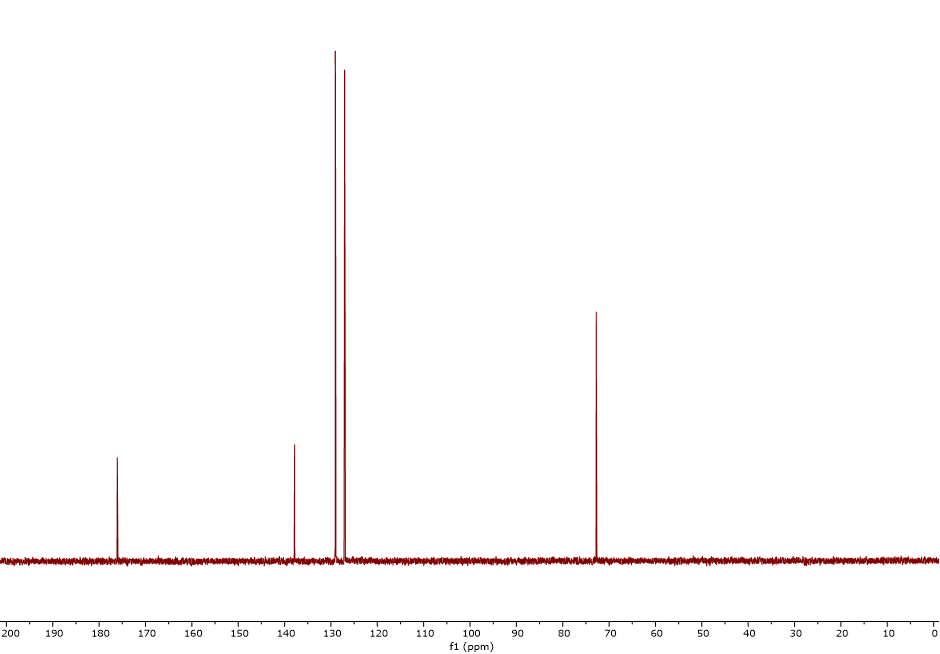
b**

**Figure S8 ^13^C NMR spectra of purified bioproduced (*S*)-MA (a) and standard (*S*)-MA (b).** Samples were dissolved in D_2_O (30 mg/mL).

**a**

**b**

**Figure S9 ^1^H NMR spectra of** **the bioproduct of (*R*)- and (*S*)-MA (a and b, respectively) before the final step of purification.** Samples were dissolved in D_2_O (30 mg/mL).

**a**

**b**

**Figure S10 ^13^C NMR spectra of the bioproduct of (*R*)- and (*S*)-MA (a and b, respectively) before the final step of purification.** Samples were dissolved in D_2_O (30 mg/mL).

**Figure S11 HPLC analysis recording the chromatogram of the purified fermentation-derived (*R*)-MA.**


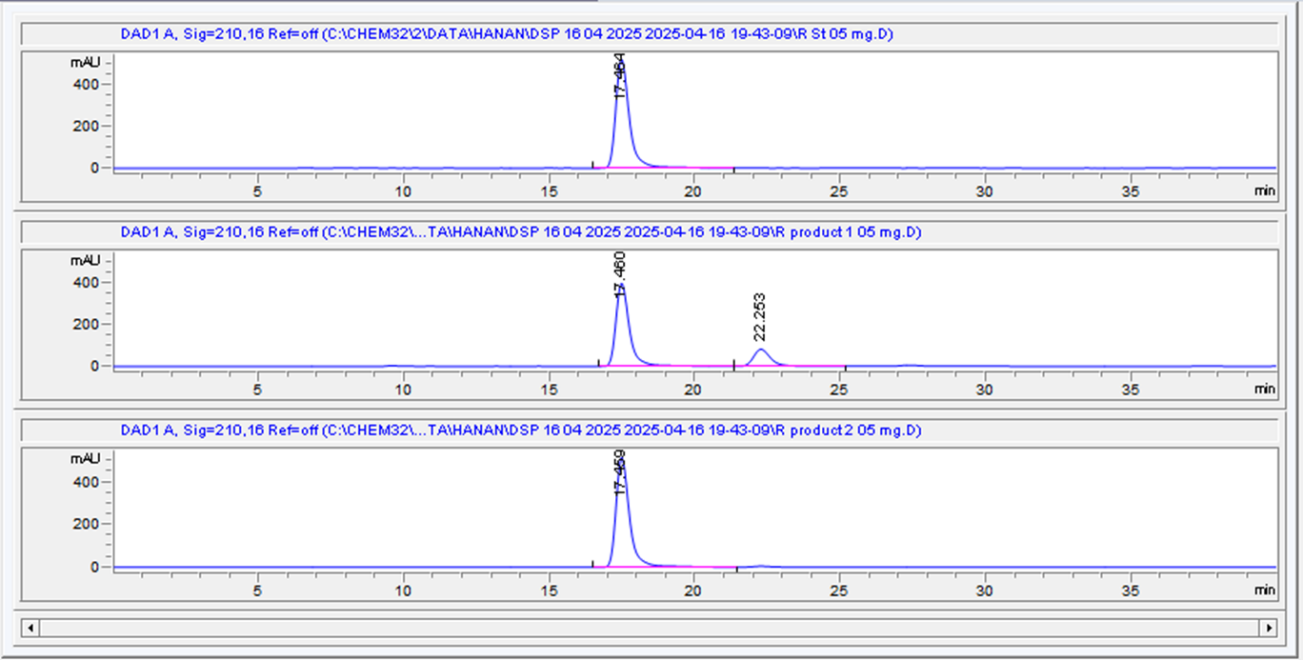


**a**

**B**

**C**

a: chromatogram of standard (*R*)-MA, b: crude (*R*)-MA product (before final purification step), c: purified (*R*)-MA. Note: The chromatograms shown in this figure were acquired on a different Agilent 1260 Infinity II HPLC system than the one used for Fig. S1. Differences in detector and pump configuration between the two systems result in a shift in absolute retention times. However, within each run, both the MA standard and the corresponding samples were analysed together, and their retention times were identical under the run-specific conditions.


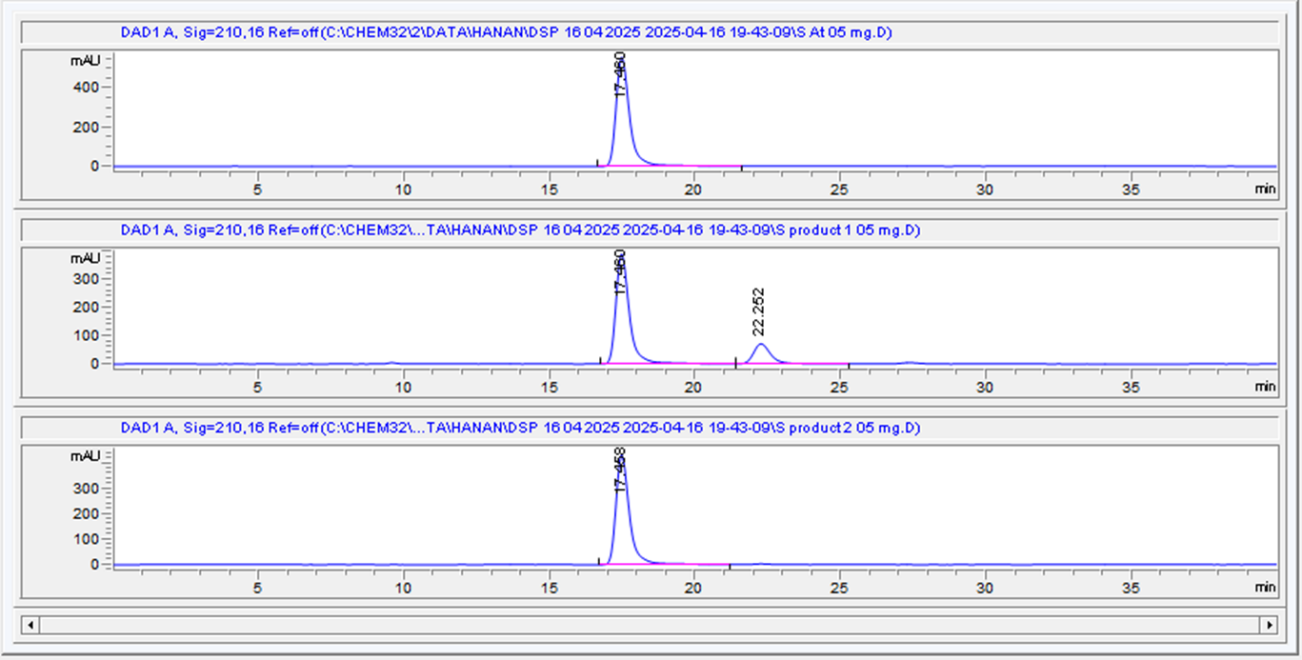


**a**

**b**

**c**

**Figure S12 HPLC analysis recording the** **chromatogram of the purified fermentation-derived (*S*)-MA.**

a: chromatogram of standard (*S*)-MA, b: crude (*S*)-MA product (before final purification step), c: purified (*S*)-MA. Note: The chromatograms shown in this figure were acquired on a different Agilent 1260 Infinity II HPLC system than the one used for Fig. S1. Differences in detector and pump configuration between the two systems result in a shift in absolute retention times. However, within each run, both the MA standard and the corresponding samples were analysed together, and their retention times were identical under the run-specific conditions.


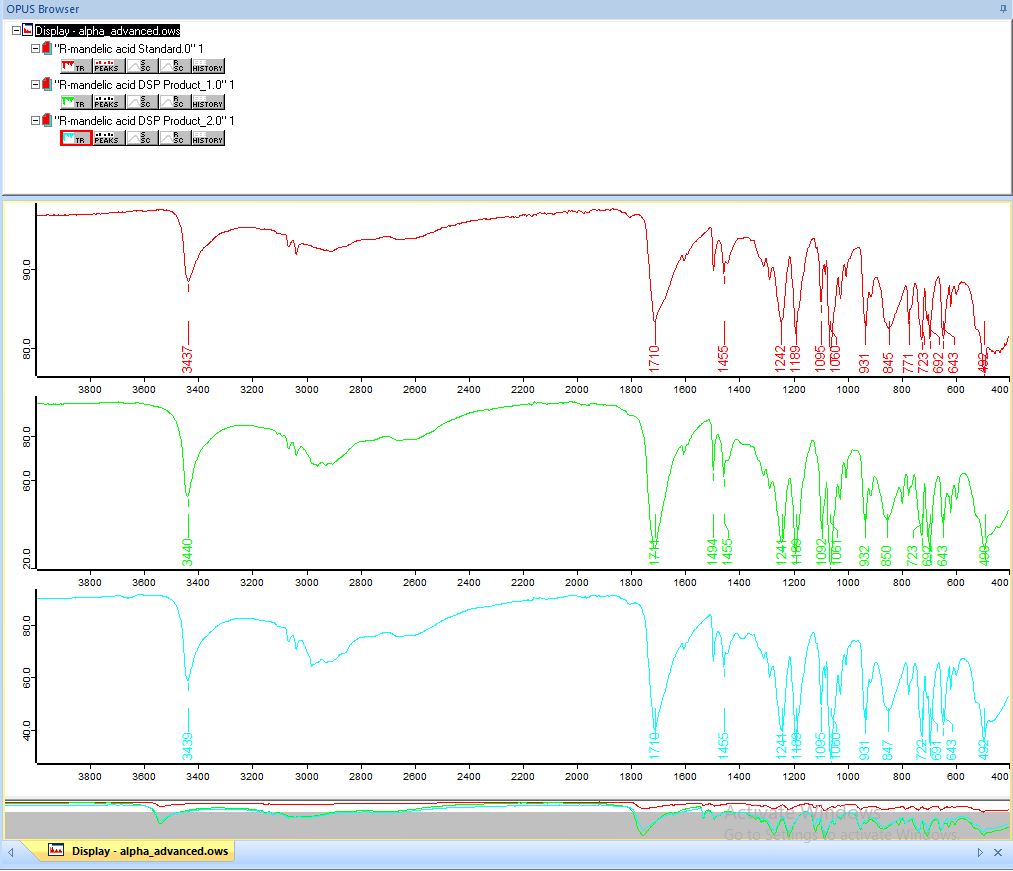


**a**

**b**

**c**

**Figure S13 IR spectra of the purified fermentation-derived (*R*)-MA.** a: standard (*R*)-MA, b: (*R*)-MA product (before final purification step), c: purified (*R*)-MA.


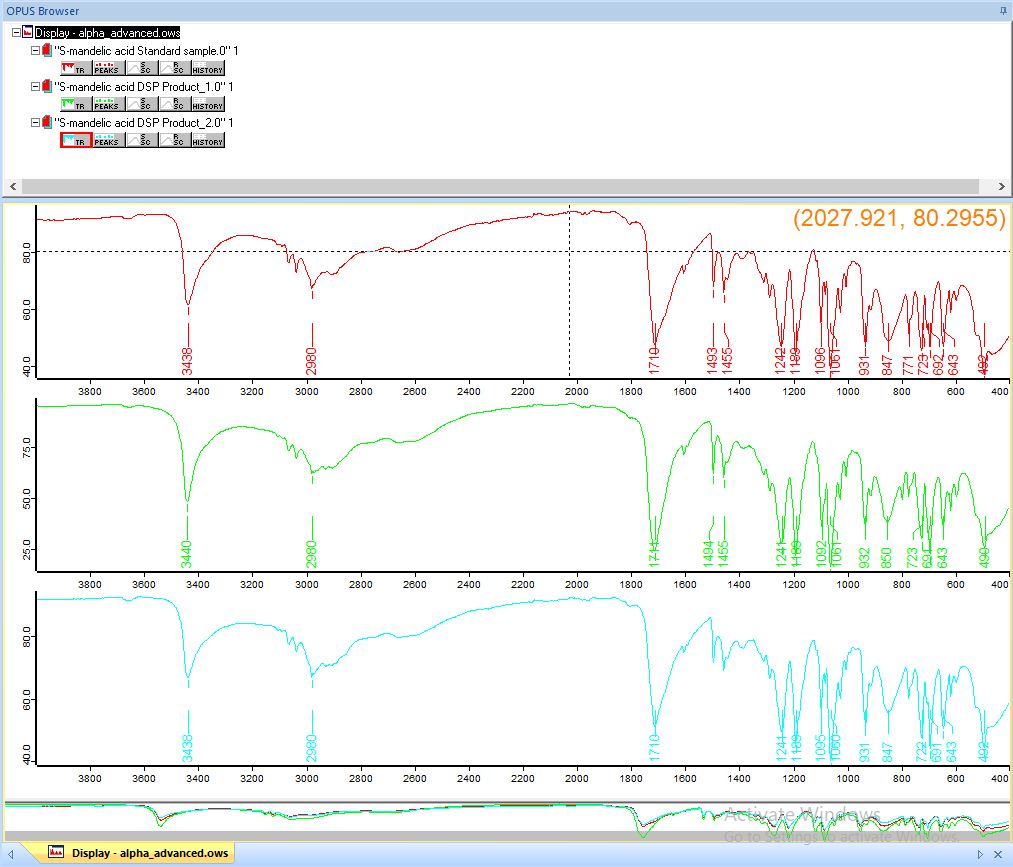


**a**

**b**

**c**

**Figure S14 IR spectra of the purified fermentation-derived (*S*)-MA.** a: standard (*S*)-MA, b: (*S*)-MA product (before final purification step), c: purified (*S*)-MA.

**Figure S15 Mass Spectrum of SAMMA synthesised from standard (*R*)-MA. (0.5 g starting material).**

**Figure S16 Mass Spectrum of SAMMA synthesised from the bioproduced (*R*)-MA. (0.5 g starting material).**

**Figure S17 Mass Spectrum of SAMMA synthesised from standard (*S*)-MA. (0.5 g starting material).**

**Figure S18 Mass Spectrum of SAMMA synthesised from the bioproduced (*S*)-MA.** **(0.5 g starting material).**

**Figure S19 Mass Spectrum of SAMMA synthesised from standard MA** **(5 g starting material).**

**Figure S20 ^1^H NMR spectrum of (*R*), (*R*)-mandelide in CDCl_3_.**

^1^H NMR (500 MHz, CDCl_3_): δ 6.14 (s, 1H), 7.28−7.47 (m, 5H). Peak at 7.26 is the CDCl_3_ solvent peak. Spectra are matching those reported previously (7).

**
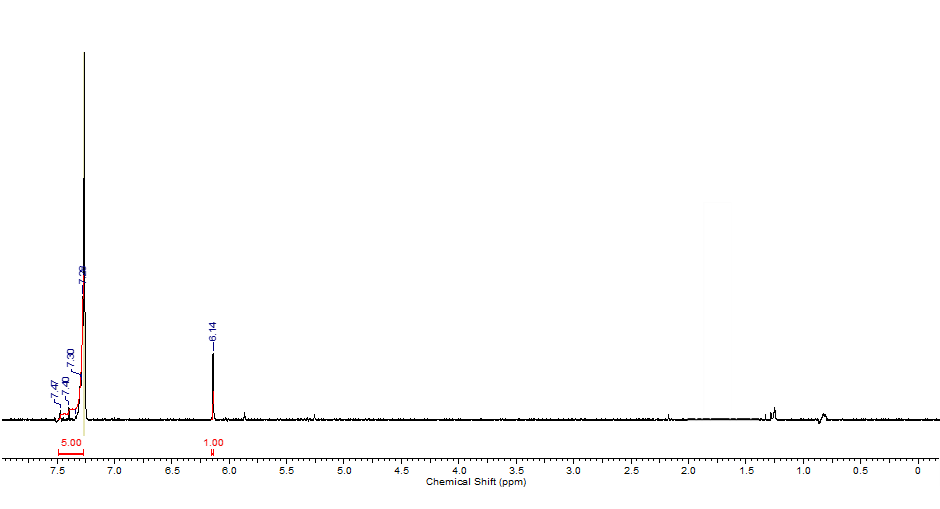
**

**Figure S21 ^1^H NMR spectrum of (*S*), (*S*)-mandelide in CDCl_3_.**

^1^H NMR (500 MHz, CDCl_3_): δ 6.14 (s, 1H), 7.28−7.47 (m, 5H). Peak at 7.26 is the CDCl_3_ solvent peak. Spectra are matching those reported previously (7).

**References:**

1. Robinson CJ, Carbonell P, Jervis AJ, Yan C, Hollywood KA, Dunstan MS, et al. Rapid prototyping of microbial production strains for the biomanufacture of potential materials monomers. Metab Eng. 2020;60:168–82.

2. Hou Y, Hossain GS, Li J, Shin HD, Du G, Liu L. Combination of phenylpyruvic acid (PPA) pathway engineering and molecular engineering of *l*-amino acid deaminase improves PPA production with an *Escherichia coli* whole-cell biocatalyst. Appl Microbiol Biotechnol. 2016;100(5):2183–91.

3. Swainston N, Dunstan M, Jervis AJ, Robinson CJ, Carbonell P, Williams AR, et al. PartsGenie: an integrated tool for optimizing and sharing synthetic biology parts. Bioinforma Oxf Engl. 2018;34(13):2327–9.

4. Jiang Y, Chen B, Duan C, Sun B, Yang J, Yang S. Multigene editing in the *Escherichia coli* genome via the CRISPR-Cas9 system. Appl Environ Microbiol. 2015;81(7):2506–14.

5. Jervis AJ, Hanko EKR, Dunstan MS, Robinson CJ, Takano E, Scrutton NS. A plasmid toolset for CRISPR-mediated genome editing and CRISPRi gene regulation in *Escherichia coli*. Microb Biotechnol. 2021;14(3):1120–9.

6. Jiang Y, Chen B, Duan C, Sun B, Yang J, Yang S. Multigene editing in the *Escherichia coli* genome via the CRISPR-Cas9 system. Appl Environ Microbiol. 2015;81(7):2506–14.

7. Graulus GJ, Van Herck N, Van Hecke K, Van Driessche G, Devreese B, Thienpont H, et al. Ring opening copolymerisation of lactide and mandelide for the development of environmentally degradable polyesters with controllable glass transition temperatures. React Funct Polym. 2018;128:16–23.
